# A Multi-Level miRNA Regulatory Network Associated with IRF1 Expression in Non-Small Cell Lung Cancer: In Silico Identification of Candidate Biomarkers for Immunotherapy Response

**DOI:** 10.3390/ijms27125192

**Published:** 2026-06-08

**Authors:** Dariya V. Karaseva, Alina M. Perevalova, Tatiana S. Kalinina, Vladislav V. Kononchuk, Vadim V. Kozlov, Lyudmila F. Gulyaeva, Vladimir O. Pustylnyak

**Affiliations:** 1 Institute for the Medicine and Medical Technology, Novosibirsk State University, Pirogova Street, 1, 630090 Novosibirsk, Russia; d.karaseva@g.nsu.ru (D.V.K.); a.perw@yandex.ru (A.M.P.); gulyaeva@niimbb.ru (L.F.G.); 2 Institute of Molecular Biology and Biophysics, Federal Research Center of Fundamental and Translational Medicine, Timakova Street, 2/12, 630117 Novosibirsk, Russia; tskalinina@frcftm.ru (T.S.K.); cvt.vvk@gmail.com (V.V.K.); vadimkozlov80@mail.ru (V.V.K.)

**Keywords:** IRF1, microRNA, non-small cell lung cancer, immune checkpoint inhibitors, PD-L1, biomarker, miR-301b, miR-183, miR-141, NFKB1, STAT4

## Abstract

Immune checkpoint inhibitors (ICIs) have revolutionized the treatment of advanced non-small cell lung cancer (NSCLC). However, the limited predictive value of PD-L1 expression as a biomarker underscores the urgent need for more reliable predictors of ICI response. Interferon regulatory factor 1 (IRF1) is a transcription factor that lies downstream of interferon-γ signaling and directly regulates *CD274* (PD-L1) transcription. Here, we performed a comprehensive bioinformatic analysis to identify microRNAs (miRNAs) that may be associated with *IRF1* expression in lung adenocarcinoma (LUAD) and lung squamous cell carcinoma (LUSC). Using data from The Cancer Genome Atlas (TCGA), we identified 20 miRNAs whose expression levels consistently and negatively correlated with IRF1 mRNA levels in both LUAD and LUSC. Among these, only hsa-miR-301b possesses conserved binding sites in the 3′UTR of IRF1 mRNA, suggesting direct post-transcriptional repression. For the remaining 19 miRNAs, we hypothesized an indirect mechanism of action. Further analysis revealed that hsa-miR-183 and hsa-miR-141 may target the transcription factor genes *NFKB1* and *STAT4*, respectively, both of which positively correlate with *IRF1* expression and are themselves associated with improved survival in ICI-treated patients. This study delineates a multi-layer miRNA regulatory network associated with *IRF1* expression in NSCLC and identifies hsa-miR-301b, hsa-miR-183 and hsa-miR-141 as candidate upstream regulators of *IRF1*. Direct survival analysis for these miRNAs in ICI-treated cohorts was not feasible due to the lack of publicly available miRNA-seq data with treatment annotations; therefore, their clinical predictive value remains hypothetical, and experimental validation is required to assess their potential as predictors of ICI response.

## 1. Introduction

Lung cancer remains the leading cause of cancer-related mortality worldwide. Non-small cell lung cancer (NSCLC) accounts for approximately 85% of all lung cancer cases and is frequently diagnosed at advanced stages, where therapeutic options are limited. The introduction of immune checkpoint inhibitors (ICIs) targeting programmed cell death protein-1 (PD-1) and its ligand PD-L1 has substantially improved outcomes for a subset of patients with advanced NSCLC. However, the clinical benefit of ICI therapy is highly heterogeneous: objective response rates range from 20% to 40% in unselected patient populations, and a substantial proportion of patients experience primary or acquired resistance [1,2].

Currently, tumor PD-L1 expression assessed using immunohistochemistry remains the most widely used biomarker for guiding ICI therapy in NSCLC. Patients with high PD-L1 expression (tumor proportion score ≥50%) are more likely to benefit from first-line ICI monotherapy. Nevertheless, the predictive accuracy of PD-L1 is imperfect. Some patients with high PD-L1 expression do not respond to ICI treatment, whereas a fraction of patients with low or even negative PD-L1 expression derive durable clinical benefit [3,4]. These observations indicate that PD-L1 is an inducible rather than a constitutive marker, and its level can vary over time depending on the dynamic tumor microenvironment [5]. There is therefore an urgent need for additional biomarkers that more reliably predict ICI response.

Interferon regulatory factor 1 (IRF1) is a transcription factor that plays a central role in the cellular response to interferons. Upon stimulation with interferon-γ (IFN-γ), the JAK-STAT signaling pathway becomes activated, leading to transcriptional induction of *IRF1* [6]. IRF1 then binds directly to the promoter of the *CD274* gene (encoding PD-L1) and drives its expression [7]. In melanoma cells, IRF1 binding to the *CD274* promoter has been demonstrated to be essential for IFN-γ-induced PD-L1 upregulation [8]. Importantly, the expression of *IRF1* is itself inducible and can be generated de novo in response to various stimuli, making it a potential “functional” biomarker that reflects the capacity of tumor cells to upregulate PD-L1 when exposed to an appropriate microenvironmental signal [9].

Recent studies have suggested that IRF1 expression may serve as a predictive biomarker for ICI response across multiple tumor types. In metastatic melanoma, high nuclear IRF1 expression in tumor tissue was associated with a durable radiographic response to anti-PD-1 therapy [10]. In NSCLC, bioinformatic analyses have shown that high IRF1 expression correlates with a favorable prognosis in patients receiving ICI treatment [11,12]. Furthermore, we previously reported that smoking-mediated downregulation of the *IRF1* gene via miR-301a may contribute to immune evasion in lung squamous cell carcinoma [13]. These observations collectively point to IRF1 as a promising candidate biomarker for ICI response.

MicroRNAs (miRNAs) are small non-coding RNA molecules that regulate gene expression post-transcriptionally by binding to complementary sequences in the 3′UTR of target mRNAs. Several miRNAs, including members of the miR-200 family, miR-21, miR-155, miR-34a, and miR-125a-3p, have been associated with immunotherapy response in lung cancer [14,15,16,17,18,19,20]. However, the molecular mechanisms by which miRNAs control key immune regulators remain incompletely understood. Given that IRF1 is an inducible factor whose cellular levels are tightly controlled, we reasoned that miRNAs capable of modulating IRF1 expression might serve as upstream regulators of the PD-L1 axis and, consequently, as biomarkers of ICI sensitivity. In this study, we performed a comprehensive bioinformatic analysis to identify miRNAs whose expression levels correlate with *IRF1* in two major histological subtypes of NSCLC—lung adenocarcinoma (LUAD) and lung squamous cell carcinoma (LUSC)—with the goal of identifying candidate miRNA biomarkers for ICI response.

## 2. Results

### 2.1. IRF1 Expression Is Reduced in LUAD and LUSC Tumor Tissues

We first examined *IRF1* expression levels across various cancer types using TIMER and TNMplot. Notably, both platforms showed significantly lower IRF1 mRNA levels in LUAD and LUSC tumor tissues compared with adjacent normal lung tissue (Supplementary Figure S1). Analysis using UALCAN confirmed these findings: IRF1 mRNA was significantly reduced in both LUAD (*p* < 0.05) and LUSC (*p* < 0.05) tumor samples relative to normal controls. The analysis included 515 tumor samples and 59 adjacent normal samples for LUAD, and 503 tumor samples and 52 adjacent normal samples for LUSC. All comparisons between tumor and normal tissues were unpaired (samples from different individuals). Protein-level analysis using CPTAC data revealed that IRF1 protein was also significantly decreased in LUAD tumor tissues (111 tumor vs. 111 normal samples, unpaired), while the reduction in LUSC (100 tumor vs. 31 normal samples, unpaired) did not reach statistical significance.

**Figure 1:**
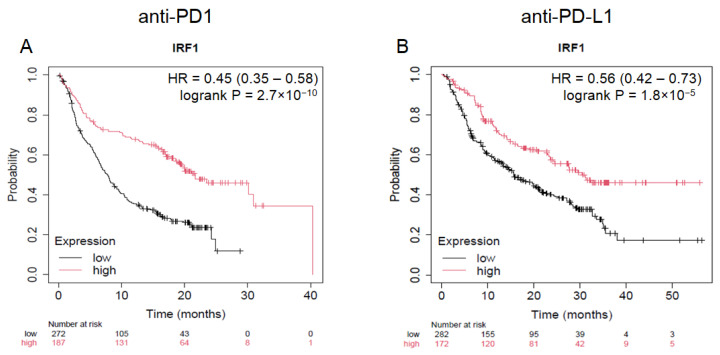
High *IRF1* expression is associated with improved survival in ICI-treated patients. Kaplan–Meier survival curves comparing patients with high versus low *IRF1* expression in cohorts receiving anti-PD-1 (**A**) or anti-PD-L1 (**B**) immunotherapy. High *IRF1* expression correlated with significantly longer overall survival (log-rank *p* < 0.05).

Kaplan–Meier survival analysis of NSCLC patients treated with ICIs using the KM Plotter platform showed that high *IRF1* expression was significantly associated with improved overall survival (Figure 1). This observation supports the hypothesis that *IRF1* may function primarily as a predictive biomarker for ICI response.

### 2.2. IRF1 Is Associated with Immune-Related Pathways in NSCLC

To understand the biological processes in which *IRF1* participates in NSCLC, we performed a correlation analysis to identify genes whose expression levels correlate with *IRF1* in LUAD and LUSC. Using the LinkedOmics platform, we identified a large number of genes significantly correlated with *IRF1* (r > 0.5) in both tumor types (FDR < 0.01). The top positively correlated genes included immune-related transcripts such as *GBP1*, *GBP4*, *PSMB9*, *TAP1*, *CCL5*, *GZMA*, *GZMB*, *PRF1*, and *CD274* itself (Supplementary Tables S1 and S2).

**Figure 2:**
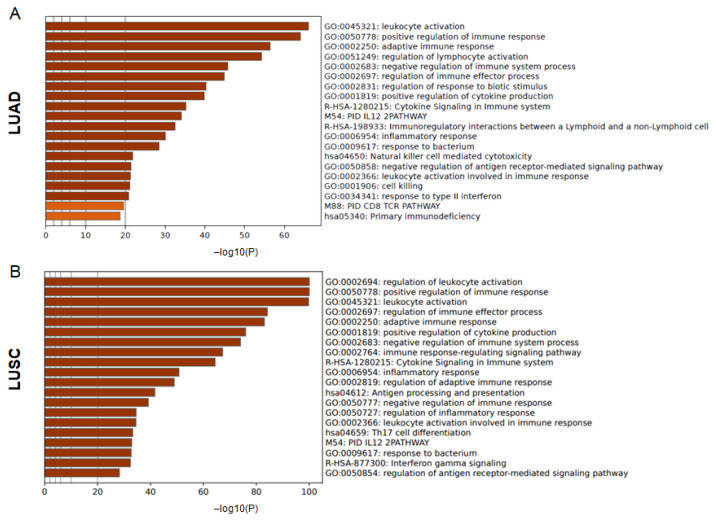
IRF1-associated genes are enriched in immune-related pathways. Bar plot of enriched GO terms for genes positively correlated with IRF1 in LUAD (**A**) and LUSC (**B**). Bar plots show the top 20 most significantly enriched terms. The colour gradient (from light brown to dark brown) represents the statistical significance, with darker brown indicating higher significance.

GSEA of these correlated genes revealed strong enrichment of immune-related pathways in both LUAD and LUSC (Figure 2). Cancer hallmark analysis using the Cancer Hallmarks Analytics Tool showed that genes associated with *IRF1* were significantly enriched for the “immune evasion” hallmark in both LUAD and LUSC, as well as for “sustained proliferative signaling” and “resistance to cell death” (Supplementary Figure S2).

### 2.3. IRF1 Expression Positively Correlates with Immune Cell Infiltration and ICI Targets

**Figure 3:**
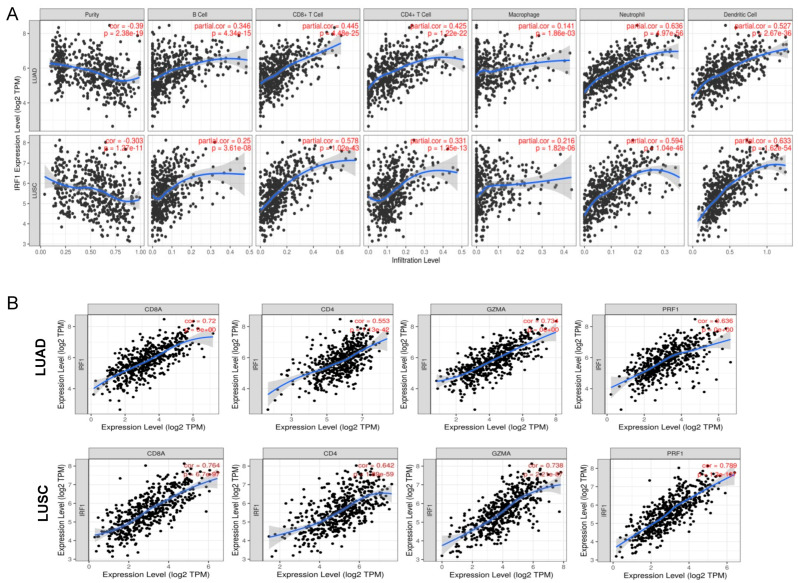
Correlation of *IRF1* expression with immune cell infiltration and cytotoxic T-cell markers in LUAD (*n* = 515) and LUSC (*n* = 501). (**A**) Spearman correlation coefficients between *IRF1* expression and the estimated abundance of six immune cell types (B cells, CD4+ T cells, CD8+ T cells, neutrophils, macrophages, dendritic cells) as determined by the TIMER platform. (**B**) Scatter plots showing pairwise correlations between *IRF1* expression and the cytotoxic T-cell markers *CD8A* (CD8+ T cells), *CD4* (CD4+ T helper cells), *GZMA* (granzyme A), and *PRF1* (perforin). Each black dot represents an individual tumor sample. The blue line indicates the best-fit linear regression line, illustrating the overall trend of the association between the two variables. The shaded area around the line represents the 95% confidence interval for the regression, indicating the level of uncertainty in the estimated trend. Spearman correlation coefficients and *p*-values are indicated on each plot. All correlations shown are statistically significant (*p* < 0.001).

Using the TIMER platform, we examined the relationship between *IRF1* expression and immune cell infiltration in LUAD and LUSC. In both histological subtypes, *IRF1* expression was significantly positively correlated with the infiltration of CD8+ T cells, CD4+ T cells, B cells, neutrophils, and dendritic cells (Figure 3A). Notably, the strongest correlations were observed for CD8+ T cells (LUAD: r = 0.445, *p* < 0.001; LUSC: r = 0.578, *p* < 0.001) and CD4+ T cells (LUAD: r = 0425, *p* < 0.001; LUSC: r = 0.331 *p* < 0.001).

We next examined the correlation between *IRF1* and markers of cytotoxic T-cell function. *IRF1* expression showed a strong positive correlation with *CD8A* (a marker of cytotoxic CD8+ T cells, LUAD: r = 0.720, *p* < 0.001; LUSC: r = 0.760, *p* < 0.001), *CD4* (LUAD: r = 0.553, *p* < 0.001; LUSC: r = 0.642, *p* < 0.001), *GZMA* (granzyme A, LUAD: r = 0.734, *p* < 0.001; LUSC: r = 0.738, *p* < 0.001), and *PRF1* (perforin, LUAD: r = 0.636, *p* < 0.001; LUSC: r = 0.789, *p* < 0.001) in both LUAD and LUSC (Figure 3B). Given that GZMA and PRF1 are key cytolytic effectors whose expression is sharply upregulated upon CD8+ T-cell activation, these findings demonstrate a positive correlation between *IRF1* expression and an activated cytotoxic T-cell phenotype.

**Figure 4:**
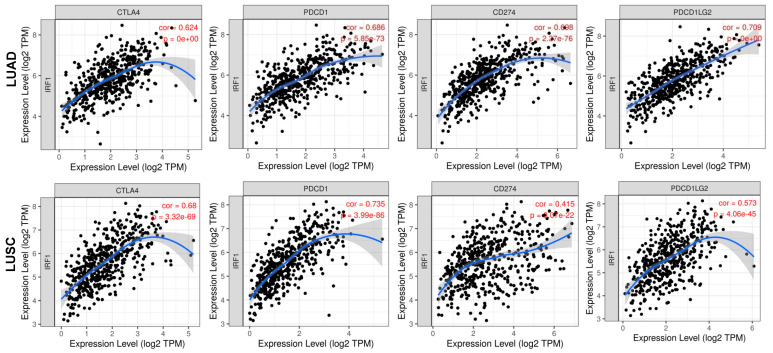
Correlation of *IRF1* expression with genes encoding major immune checkpoint targets in LUAD (*n* = 515) and LUSC (*n* = 501). Scatter plots show pairwise correlations between *IRF1* expression and *CTLA4*, *PDCD1* (encoding PD-1), *CD274* (encoding PD-L1), and *PDCD1LG2* (encoding PD-L2) in LUAD (**upper panels**) and LUSC (**lower panels**). Each black dot represents an individual tumor sample. The blue line indicates the best-fit linear regression line, illustrating the overall trend of the association between the two variables. The shaded area around the line represents the 95% confidence interval for the regression, indicating the level of uncertainty in the estimated trend. Spearman correlation coefficients and *p*-values are shown on each plot. All correlations are statistically significant (*p* < 0.001).

Furthermore, using the TIMER platform, we analyzed the correlation between *IRF1* and the genes encoding major ICI targets. *IRF1* expression was significantly positively correlated with *CTLA4* (LUAD: r = 0.624, *p* < 0.001; LUSC: r = 0.680, *p* < 0.001), *PDCD1* (encoding PD-1, LUAD: r = 0.686, *p* < 0.001; LUSC: r = 0.735, *p* < 0.001), *CD274* (encoding PD-L1, LUAD: r = 0.608, *p* < 0.001; LUSC: r = 0.415, *p* < 0.001), and *PDCD1LG2* (encoding PD-L2, LUAD: r = 0.709, *p* < 0.001; LUSC: r = 0.573, *p* < 0.001) in both LUAD and LUSC (Figure 4). The positive correlation with *CD274* is consistent with the known role of IRF1 as a direct transcriptional activator of PD-L1.

### 2.4. Identification of miRNAs Negatively Correlated with IRF1 in LUAD and LUSC

**Figure 5:**
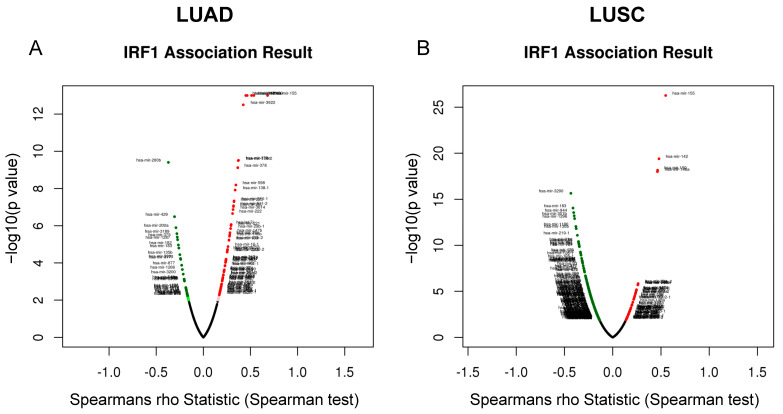
Identification of miRNAs negatively correlated with *IRF1* in LUAD (*n* = 515) and LUSC (*n* = 501). Volcano plots showing miRNAs significantly positively (red) and negatively (green) correlated with IRF1 in LUAD (**A**) and LUSC (**B**). Black dots represent miRNAs that did not reach statistical significance (FDR ≥ 0.01).

To identify miRNAs that may regulate *IRF1* expression, we performed correlation analysis between IRF1 mRNA levels and miRNA expression in LUAD and LUSC using LinkedOmics. Volcano plots revealed a substantial number of miRNAs significantly negatively correlated with *IRF1* in both lung tumor types (FDR < 0.01) (Figure 5). From these, we selected miRNAs with the strongest negative correlation coefficients for further analysis (LUAD, Supplementary Table S3; LUSC, Supplementary Table S4).

From all miRNAs showing a statistically significant negative correlation (FDR < 0.01), we selected those with the strongest negative association using a threshold of Spearman r < −0.15. To identify miRNAs relevant to both histological subtypes, we applied an intersection criterion: a miRNA had to meet the selection criteria (FDR < 0.01 and r < −0.15) in both LUAD and LUSC simultaneously. This yielded 20 shared miRNAs, which are listed in Table 1. Among these shared miRNAs were several members of the miR-200 family (miR-200a, miR-200b, miR-200c, miR-141, miR-429), as well as miR-182, miR-183, miR-96, and miR-301b.

### 2.5. Only hsa-miR-301b Has Conserved Binding Sites in the IRF1 3′UTR

We next investigated whether the 20 identified miRNAs might directly target IRF1 mRNA. Using TargetScan and miRDB, we searched for conserved miRNA binding sites in the 3′UTR of IRF1. Among the 20 miRNAs, only hsa-miR-301b was predicted to have conserved binding sites. Specifically, TargetScan identified two conserved 7-mer sites in the IRF1 3′UTR: one 7mer-1A site at positions 395–401 (context++ score percentile = 92%) and one 8mer site at positions 410–417 (context++ score percentile = 99%) (Table 2). These high percentile scores indicate strong evolutionary conservation and favorable targeting efficacy. For the remaining 19 miRNAs, no conserved binding sites in the IRF1 3′UTR were identified, suggesting that their negative correlation with *IRF1* may be indirect.

### 2.6. hsa-miR-301b Is Upregulated in LUAD and LUSC Tumor Tissues

**Figure 6:**
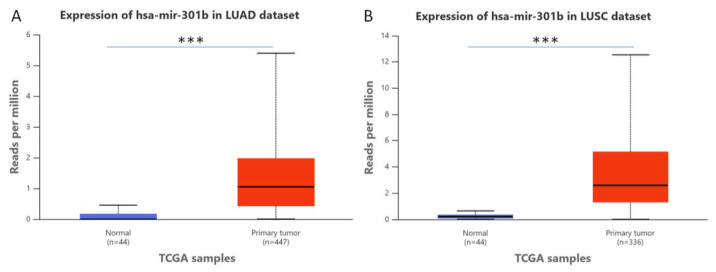
hsa-miR-301b is upregulated in LUAD and LUSC. Boxplots showing hsa-miR-301b expression in tumor versus normal tissues (*n* = 44) in LUAD ((**A**), *n* = 447) and LUSC ((**B**), *n* = 336). *** *p* < 0.001 (UALCAN).

Given its potential as a direct regulator of *IRF1*, we examined the expression of hsa-miR-301b in LUAD and LUSC using UALCAN. Compared with adjacent normal lung tissue, hsa-miR-301b expression was significantly elevated in both LUAD and LUSC tumor samples (Figure 6). This upregulation is consistent with the negative correlation observed between miR-301b and *IRF1*, as increased hsa-miR-301b levels would be expected to suppress *IRF1* expression.

### 2.7. Indirect Regulation of IRF1 via Transcription Factor-Targeting miRNAs

For the 19 miRNAs that lack direct binding sites in the IRF1 3′UTR, we hypothesized that they might regulate *IRF1* indirectly by targeting transcription factors that control *IRF1* transcription. To test this hypothesis, we first identified transcription factors known to regulate *IRF1* expression using the TRRUST database. We identified eight transcription factors with experimentally validated roles in *IRF1* regulation: CIITA, CREBBP, NFKB1, RELA, STAT1, STAT2, STAT3, and STAT4 (Table 3). We then examined the correlation between the transcription factor genes and *IRF1* in LUAD and LUSC using TIMER. As shown in Table 3, the majority of these factors showed significant positive correlations with *IRF1* in both histological subtypes, consistent with their activating roles. The strongest correlations were observed for *STAT1* (LUAD: r = 0.750, *p* < 0.001; LUSC: r = 0.770, *p* < 0.001) and *STAT4* (LUAD: r = 0.470, *p* < 0.001; LUSC: r = 0.550, *p* < 0.001). *NFKB1* also showed moderate but significant positive correlations (LUAD: r = 0.450, *p* < 0.001; LUSC: r = 0.390, *p* < 0.001). In contrast, *CREBBP* showed no significant correlation with *IRF1* in LUSC, and *STAT3* showed only weak correlations, particularly in LUAD. These results suggest that among the nine transcription factors, STAT1, STAT4, and NFKB1 are the most relevant for *IRF1* regulation in both subtypes of NSCLC.

Next, we assessed whether any of the 19 miRNAs that lack direct IRF1 binding sites might instead target these transcription factors. Using TargetScan and miRDB, we identified that hsa-miR-183 is predicted to target *NFKB1*, and hsa-miR-141 is predicted to target *STAT4*. Correlation analysis using LinkedOmics confirmed that miR-183 expression is significantly negatively correlated with *NFKB1* in both LUAD and LUSC (Table 4). Similarly, miR-141 expression is significantly negatively correlated with *STAT4* in both LUAD and LUSC (Table 4). No miRNA among the 19 showed conserved binding sites for the *STAT1* transcription factor.

We then investigated whether *NFKB1* and *STAT4* expression correlates with *CD274* (PD-L1) expression, given that both factors are known to regulate *IRF1*, which in turn drives *CD274* transcription. Correlation analysis using the TIMER platform revealed that *NFKB1* expression was significantly positively correlated with *CD274* in both LUAD (r = 0.464, *p* < 0.001) and LUSC (r = 0.214, *p* < 0.001; Supplementary Figure S3A). Similarly, *STAT4* expression showed a significant positive correlation with *CD274* in both LUAD (r = 0.480, *p* < 0.001) and LUSC (r = 0.254, *p* < 0.001; Supplementary Figure S3B). These positive correlations are consistent with a potential regulatory cascade, suggesting that NFKB1 and STAT4 may influence *IRF1* transcription and, consequently, *CD274* expression.

**Figure 7:**
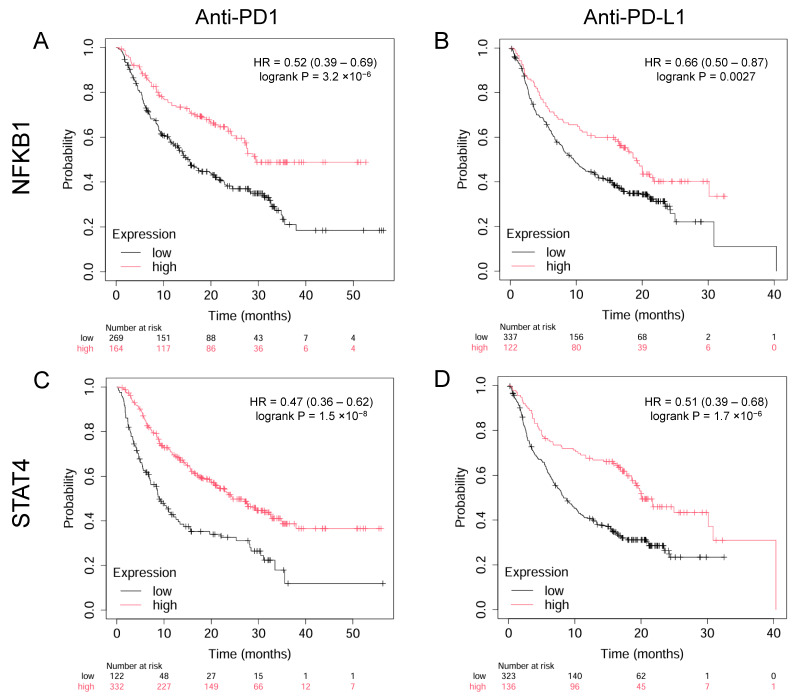
High NFKB1 and STAT4 expression is associated with improved survival in ICI-treated patients. Kaplan–Meier survival curves for NFKB1 (**A**,**B**) and STAT4 (**C**,**D**) in patients receiving anti-PD-1 (**A**,**C**) or PD-L1 (**B**,**D**) immunotherapy.

Finally, we examined whether *NFKB1* and *STAT4* expression levels are associated with ICI response. Using the KM Plotter platform, we found that high expression of either *NFKB1* or *STAT4* was significantly associated with improved overall survival in ICI-treated patient cohorts (Figure 7). This observation parallels the association we observed for *IRF1* itself and supports the clinical relevance of this regulatory axis.

## 3. Discussion

The transcription factor IRF1 has emerged as a central node in the cellular response to interferons and as a direct transcriptional activator of *CD274* (PD-L1) [7,8]. In the context of immune checkpoint blockade, IRF1 expression has been associated with favorable outcomes in melanoma and NSCLC [10,11,12]. Our bioinformatic analysis confirms that high *IRF1* expression is linked to improved survival specifically in ICI-treated patients. This pattern supports the view that *IRF1* functions as a predictive biomarker of ICI sensitivity.

The mechanistic rationale for IRF1 as a predictive marker lies in its ability to drive PD-L1 expression upon IFN-γ stimulation. Tumors that retain the capacity to upregulate PD-L1 via an intact IRF1 axis are more likely to respond to PD-1/PD-L1 blockade [21]. Our observations of positive correlations between *IRF1* and *CD274*, as well as with markers of CD8+ T-cell infiltration and cytolytic activity (*GZMA*, *PRF1*), are consistent with this model. Conversely, loss or suppression of IRF1 would compromise the ability to mount a PD-L1-mediated adaptive immune resistance, potentially leading to primary resistance to ICIs.

Given that IRF1 is an inducible protein whose levels are tightly regulated, we sought to identify upstream miRNAs that may control its expression. We performed a comprehensive bioinformatic analysis to identify miRNAs that may regulate *IRF1* expression in NSCLC. Among 20 miRNAs that consistently correlate negatively with *IRF1* in both LUAD and LUSC, only hsa-miR-301b possesses conserved binding sites in the IRF1 3′UTR, suggesting that it may directly repress *IRF1* expression. This finding is consistent with previous reports that miR-301b functions as an oncogene in lung cancer, promoting cell proliferation and inhibiting apoptosis [22,23]. Moreover, our findings are strongly supported by the work of Qi et al., who independently identified miR-301b-3p as a potential diagnostic marker for LUAD using TCGA data and machine learning approaches [24]. In their study, overexpression of miR-301b-3p accelerated tumor growth in a mouse xenograft model, while its downregulation impeded LUAD progression. Importantly, Qi et al. also performed tumor immunity and mutation analyses, finding that patients in the low-miR-301b-3p expression group had better immune infiltration, suggesting that their response rates to immunotherapy may be relatively high [24]. This observation is entirely consistent with our proposed model, in which high hsa-miR-301b levels suppress IRF1, leading to reduced immune infiltration and a potentially poorer ICI response.

For the remaining miRNAs, our data support an indirect mechanism: they may regulate the expression of transcription factors that control *IRF1* transcription. In particular, we present evidence that hsa-miR-183 may target *NFKB1* and hsa-miR-141 may target *STAT4*, both of which are positive regulators of *IRF1* expression and are themselves associated with improved ICI response. The identification of *NFKB1* and *STAT4* as potential mediators of miR-183 and miR-141 effects, respectively, provides a plausible mechanistic explanation for their negative correlation with *IRF1*. Both NFKB1 and STAT4 are established activators of *IRF1* transcription: NFKB1 binds to the *IRF1* promoter and induces its expression in response to inflammatory signals [25], while STAT4 is a key mediator of IFN-γ signaling that activates *IRF1* transcription through GAS elements in its promoter [26]. The positive correlations we observed between *NFKB1* and *IRF1*, and between *STAT4* and *IRF1*, in both LUAD and LUSC support this model. Furthermore, the negative correlations between hsa-miR-183 and *NFKB1* and between hsa-miR-141 and *STAT4* are consistent with the predicted targeting relationships. The association of high *NFKB1* and *STAT4* expression with improved survival in ICI-treated patients mirrors the association that we observed for *IRF1* itself. This finding suggests that the entire regulatory axis—from miRNAs through transcription factors to IRF1 and ultimately to PD-L1—may be relevant for ICI response. While *IRF1*, *NFKB1* and *STAT4* show survival associations in ICI-treated cohorts, equivalent analyses for the miRNAs are not possible with current public data. Thus, their proposed role as ICI response biomarkers is based on their predicted regulatory relationships with *IRF1* and requires independent validation.

Integrating our findings, we propose a multi-layer regulatory network for IRF1 in NSCLC. At the direct level, hsa-miR-301b is predicted to bind to two conserved sites in the IRF1 3′UTR and may directly repress IRF1 translation. At the indirect level, hsa-miR-183 and hsa-miR-141 may target the transcription factors NFKB1 and STAT4, respectively. Reduced expression of these transcription factors would lead to decreased *IRF1* transcription, thereby lowering IRF1 protein levels. Lower IRF1, in turn, would compromise the ability of tumor cells to upregulate PD-L1 in response to IFN γ, potentially leading to reduced sensitivity to PD-1/PD-L1 blockade. This model is consistent with the observed positive correlations between these transcription factors, *IRF1*, and *CD274*, as well as their associations with favorable ICI outcomes. Importantly, our data indicate that this axis is largely preserved across both major histological subtypes of NSCLC (LUAD and LUSC), although the strength of individual correlations differs between LUAD and LUSC.

While the overall regulatory network was present in both histological subtypes of NSCLC, we observed notable differences in the strength of individual correlations between LUAD and LUSC. For example, the negative correlation between miR-301b and *IRF1* was much stronger in LUSC (r = −0.400, *p* < 0.001) than in LUAD (r = −0.179, *p* < 0.001). Similarly, the correlation between miR-141 and *STAT4* was markedly stronger in LUSC (r = −0.420, *p* < 0.001) compared to LUAD (r = −0.200, *p* < 0.001). Additionally, IRF1 protein levels, as assessed using CPTAC data, were significantly reduced only in LUAD, not in LUSC. These discrepancies suggest that the relative contributions of direct miRNA-mediated repression versus indirect transcription factor-mediated regulation may differ between lung adenocarcinoma and lung squamous cell carcinoma. Possible explanations include differences in the cellular origin (alveolar type II cells for LUAD vs. basal airway epithelial cells for LUSC) [27], distinct mutational landscapes [28], and divergent immune microenvironments [29]. Therefore, while the key molecular players (miR-301b, miR-183, miR-141, *NFKB1*, *STAT4*, and *IRF1*) are relevant in both subtypes, the quantitative impact of each regulatory interaction may be context-dependent. Future experimental validation should be performed separately in LUAD- and LUSC-derived cell lines to capture these subtype-specific differences.

Several limitations of this study should be acknowledged. First, all findings are derived from publicly available bulk-tissue datasets (TCGA) and computational prediction tools. Second, the proposed direct binding of hsa-miR-301b to the IRF1 3′UTR, and the indirect regulation of *IRF1* by hsa-miR-183 and hsa-miR-141 via NFKB1 and STAT4, remain speculative until validated by functional experiments (e.g., luciferase reporter assays, miRNA mimic/inhibitor transfections with measurement of IRF1 protein levels). Third, the survival analyses for ICI-treated patients were performed using the Kaplan–Meier Plotter platform, which aggregates data from multiple cohorts without providing detailed clinical annotations (e.g., PD-L1 status, line of therapy, specific ICI agent, prior treatments). Therefore, we cannot rule out confounding factors such as tumor mutational burden, smoking history, or differences in the tumor microenvironment. Fourth, because IRF1 is also expressed in immune cells, the positive correlations between *IRF1* and immune infiltration markers (*CD8A*, *GZMA*, *PRF1*) may partly reflect the presence of infiltrating lymphocytes rather than tumor-intrinsic IRF1 expression. Without single-cell or sorted-cell data, we cannot definitively attribute the observed associations to malignant cells. Fifth, the miRNA selection pipeline and survival analyses were not validated on independent external cohorts with matched miRNA-seq and ICI treatment outcome data. Finally, the manuscript does not include direct experimental evidence or patient-level validation of the proposed miRNAs as predictive biomarkers. Consequently, our findings should be interpreted as hypothesis-generating, and the proposed regulatory network requires further experimental and clinical validation.

Nevertheless, the clinical implications of our findings are potentially significant. Currently, PD-L1 expression is the only biomarker routinely used to guide ICI therapy in NSCLC, but its limitations are well-recognized. miRNAs offer several advantages as biomarkers: they are stable in formalin-fixed paraffin-embedded tissues and can be measured in minimally invasive liquid biopsies [30]. The miRNAs identified in this study—particularly miR-301b, miR-183, and miR-141—could potentially be incorporated into multi-biomarker panels to improve the prediction of ICI response, especially in patients with intermediate or low PD-L1 expression where treatment decisions are most challenging.

Thus, this bioinformatic study identifies a multi-layer miRNA regulatory network controlling IRF1 expression in NSCLC. hsa-miR-301b is predicted to directly target the IRF1 3′UTR, while hsa-miR-183 and hsa-miR-141 may indirectly regulate *IRF1* through targeting the transcription factors NFKB1 and STAT4, respectively. All three miRNAs, as well as their transcription factor targets, are associated with ICI response outcomes, suggesting that they may serve as candidate predictive biomarkers. Experimental validation of these findings is warranted.

## 4. Materials and Methods

### Bioinformatic Analysis

Differential Expression Analysis using TIMER. The TIMER platform (https://cistrome.shinyapps.io/timer/, accessed on 23 March 2026) facilitated an initial broad assessment of *IRF1* expression. TIMER automatically queried the pre-processed TCGA RNA-Seq dataset, which includes data from 32 cancer types and 10,897 samples. In the “DiffExp” module, we input IRF1 as the target gene, which automatically generated box plots illustrating its log2 RSEM expression distribution. TIMER employs the Wilcoxon rank-sum test, a non-parametric method ideal for comparing expression levels between two independent groups, such as tumor and normal samples.

Differential Expression Analysis using TNMplot. TNMplot (https://tnmplot.com/analysis/, accessed on 23 March 2026) is a robust web-based platform that integrates RNA-Seq and gene-chip data from a comprehensive collection of 56,938 samples. The platform consolidates transcriptomic data from three primary sources: the Genotype-Tissue Expression (GTEx) project for normal tissues, and both the TCGA and TARGET databases for tumor and metastatic samples. To validate TIMER findings, we utilized TNMplot with the “Normal vs. Primary Tumor” option, generating box plots with default log2 (TPM) or log2 (RSEM) scaling. The Mann–Whitney U test is utilized to determine the significance of expression differences between two groups.

mRNA Expression Analysis using UALCAN. UALCAN (http://ualcan.path.uab.edu, accessed on 23 March 2026) is a comprehensive portal that offers in-depth analysis of cancer transcriptome data. For quantifying IRF1 transcript levels, the UALCAN platform was used. The analysis is based on UALCAN’s integrated Level 3 RNA-seq data from TCGA (RSEM-normalized). The tool automatically generated box plots of log2 (TPM + 1) values. The Wilcoxon rank-sum test is the standard method used for UALCAN’s differential expression analysis.

Protein Expression Analysis using UALCAN. UALCAN (http://ualcan.path.uab.edu, accessed on 23 March 2026) facilitates validation at the proteomic level by providing access to mass spectrometry-based proteomics data from the Clinical Proteomic Tumor Analysis Consortium (CPTAC). The underlying CPTAC data undergo two-stage normalization: Log2 Spectral Count Ratio values are first normalized within each sample profile and then across all samples. The final output is reported as Z-values, which represent standard deviations from the median across all samples. The resulting box plots display the distribution of protein expression Z-values, with statistical significance assessed using the t-test.

Correlation analysis between IRF1 mRNA expression and miRNA expression was performed using the LinkFinder module of the LinkedOmics platform (http://www.linkedomics.org, accessed on 23 March 2026). The analysis was conducted separately for the TCGA LUAD and LUSC cohorts. For each cohort, we retrieved normalized expression data for *IRF1* and for all mature miRNAs. Only samples with matched mRNA and miRNA expression profiles were included. Spearman’s rank correlation coefficient was calculated for each miRNA–*IRF1* pair. Spearman’s method was chosen because it is non-parametric and robust to outliers, making it suitable for expression data that may not follow a normal distribution. To control for false positives due to multiple hypothesis testing, the Benjamini–Hochberg false discovery rate (FDR) correction was applied. An FDR <0.01 was considered statistically significant for identifying miRNAs correlated with *IRF1*. Both nominal *p*-values and FDR-adjusted q-values were recorded.

Correlation analysis between IRF1 and other genes (including immune markers, ICI targets, and transcription factors) was performed using the TIMER platform (https://cistrome.shinyapps.io/timer/, accessed on 23 March 2026). The “Correlation” module of TIMER was employed to evaluate pairwise correlations between *IRF1* and target genes of interest (e.g., *CD8A*, *CD4*, *GZMA*, *PRF1*, *CTLA4*, *PDCD1*, *CD274*, *PDCD1LG2*, *NFKB1*, *STAT4*, etc.). For each pair, the platform automatically retrieves log_2_TPM (transcripts per million) normalized expression values across all samples in the selected cohort. TIMER uses Spearman’s rank correlation coefficient to assess monotonic relationships between genes. Spearman’s method is non-parametric and robust to outliers, making it suitable for expression data that may not follow a normal distribution. The platform provides both the correlation coefficient (r) and the statistical significance (*p*-value) calculated using a two-tailed test.

Immune cell infiltration analysis was performed using TIMER, which estimates the abundance of six immune cell types (B cells, CD4+ T cells, CD8+ T cells, neutrophils, macrophages and dendritic cells) based on gene expression signatures. Correlation coefficients and *p*-values were calculated as described above.

Gene set enrichment analysis (GSEA) was carried out using Metascape (https://metascape.org, accessed on 23 March 2026) to identify biological pathways and processes associated with genes correlated with *IRF1* expression in LUAD and LUSC. For each tumor type (LUAD and LUSC separately), we used the genes most correlated with IRF1 (based on Spearman’s r from LinkedOmics analysis). Metascape integrates multiple curated gene set collections: KEGG Pathway, GO Biological Processes, Reactome Gene Sets, Canonical Pathways, CORUM, WikiPathways and PANTHER Pathway. Metascape employs the one-tailed Fisher’s exact test to determine whether the overlap between the input gene list and each gene set is statistically significant.

Cancer hallmark enrichment analysis was performed using the Cancer Hallmarks web tool (https://cancerhallmarks.com/, accessed on 23 March 2026), employing the core gene set of 1574 genes documented in at least two of seven reference sources. For each tumor type (LUAD and LUSC separately), we used the genes most correlated with *IRF1* (based on Spearman’s r from LinkedOmics analysis).

miRNA target prediction was conducted using TargetScan (release 8.0) and miRDB (https://mirdb.org/). Only conserved binding sites in the 3′UTR of target genes were considered.

Survival analysis was performed using the Kaplan–Meier Plotter platform (http://kmplot.com, accessed on 23 March 2026), which integrates gene expression data from GEO, EGA and TCGA. For analysis of ICI-treated patients, the platform’s dedicated immunotherapy dataset was used. The platform does not allow manual stratification by clinical covariates (e.g., age, sex, stage) in the immunotherapy module due to limited sample size and data availability. To avoid arbitrary threshold selection, the platform automatically determines the optimal cutoff for gene expression stratification using the following algorithm: all possible cutoff values between the lower and upper quartiles of the expression range are evaluated; for each cutoff, the log-rank test *p*-value is calculated; the false discovery rate (FDR) is then estimated using the Benjamini–Hochberg method to correct for multiple hypothesis testing; and the cutoff that yields the most significant (lowest FDR) log-rank *p*-value is selected. The Kaplan–Meier Plotter platform automatically calculates 95% confidence intervals (CIs) for the hazard ratio using the Cox proportional hazards model. These 95% CIs are reported together with the HR and the log-rank *p*-value.

Transcription factor binding information was retrieved from TRRUST (Transcriptional Regulatory Relationships Unraveled by Sentence-based Text mining), a manually curated database of experimentally validated human transcription factor–target gene relationships.

## Figures and Tables

**Table 1:** List of 20 miRNAs negatively correlated with IRF1 in both LUAD and LUSC.

miRNA	LUAD			LUSC		
	Spearman Correlation	FDR (BH)	*p*-Value	Spearman Correlation	FDR (BH)	*p*-Value
hsa-miR-200b	−0.371	3.51 × 10^−8^	3.97 × 10^−10^	−0.318	1.55 × 10^−7^	4.38 × 10^−9^
hsa-miR-429	−0.306	1.36 × 10^−5^	3.25 × 10^−7^	−0.335	2.33 × 10^−8^	5.43 × 10^−10^
hsa-miR-200a	−0.291	4.61 × 10^−5^	1.27 × 10^−6^	−0.277	7.95 × 10^−6^	3.70 × 10^−7^
hsa-miR-182	−0.265	2.69 × 10^−4^	1.08 × 10^−5^	−0.332	3.10 × 10^−8^	7.60 × 10^−10^
hsa-miR-183	−0.260	3.70 × 10^−4^	1.58 × 10^−5^	−0.412	1.22 × 10^−12^	8.97 × 10^−15^
hsa-miR-200c	−0.243	1.15 × 10^−3^	5.77 × 10^−5^	−0.245	9.97 × 10^−5^	7.69 × 10^−6^
hsa-miR-877	−0.230	2.21 × 10^−3^	1.33 × 10^−4^	−0.301	8.10 × 10^−7^	3.08 × 10^−8^
hsa-miR-3200	−0.214	5.72 × 10^−3^	4.02 × 10^−4^	−0.434	3.64 × 10^−14^	2.23 × 10^−16^
hsa-miR-548v	−0.204	9.46 × 10^−3^	7.48 × 10^−4^	−0.268	1.79 × 10^−5^	9.02 × 10^−7^
hsa-miR-96	−0.201	1.04 × 10^−2^	8.76 × 10^−4^	−0.355	2.55 × 10^−9^	4.38 × 10^−11^
hsa-miR-3065	−0.200	1.08 × 10^−2^	9.43 × 10^−4^	−0.192	3.17 × 10^−3^	4.86 × 10^−4^
hsa-miR-1180	−0.186	2.30 × 10^−2^	2.17 × 10^−3^	−0.382	7.35 × 10^−11^	9.00 × 10^−13^
hsa-miR-301b	−0.179	3.11 × 10^−2^	3.17 × 10^−3^	−0.400	6.33 × 10^−12^	6.20 × 10^−14^
hsa-miR-3127	−0.174	3.76 × 10^−2^	4.07 × 10^−3^	−0.245	9.97 × 10^−5^	7.81 × 10^−6^
hsa-miR-1276	−0.172	4.11 × 10^−2^	4.54 × 10^−3^	−0.227	3.36 × 10^−4^	3.66 × 10^−5^
hsa-miR-556	−0.168	4.75 × 10^−2^	5.55 × 10^−3^	−0.206	1.29 × 10^−3^	1.79 × 10^−4^
hsa-miR-219-1	−0.167	4.94 × 10^−2^	5.87 × 10^−3^	−0.367	5.55 × 10^−10^	8.15 × 10^−12^
hsa-miR-141	−0.164	5.75 × 10^−2^	7.00 × 10^−3^	−0.238	1.58 × 10^−4^	1.42 × 10^−5^
hsa-miR-187	−0.163	5.84 × 10^−2^	7.27 × 10^−3^	−0.280	5.91 × 10^−6^	2.68 × 10^−7^
hsa-miR-3170	−0.154	7.67 × 10^−2^	1.11 × 10^−2^	−0.261	3.00 × 10^−5^	1.72 × 10^−6^

**Table 2:** Predicted binding sites for hsa-miR-301b in the IRF1 3′UTR.

miRNA	Position in 3′UTR	Site Type	Context++ Score	Context++ Score Percentile
hsa-miR-301b-3p	395–401	7mer-1A	−0.22	92
hsa-miR-301b-3p	410–417	8mer	−0.51	99

**Table 3:** Transcription factors regulating *IRF1* gene transcription.

Transcription Factor	Type of Action	Correlation with IRF1 in LUAD	Correlation with IRF1 in LUSC
CIITA	Unknown	0.57, *p* = 1.2 × 10^−45^	0.72, *p* = 1.8 × 10^−83^
CREBBP	Activation	0.17, *p* = 6.6 × 10^−5^	0.062, *p* = 1.5 × 10^−1^
NFKB1	Activation	0.45, *p* = 1.2 × 10^−27^	0.39, *p* = 1.1 × 10^−19^
RELA	Activation	0.27, *p* = 1.6 × 10^−10^	0.16, *p* = 1.8 × 10^−4^
STAT1	Activation/Repression	0.75, *p* = 1.5 × 10^−45^	0.77, *p* = 4.9 × 10^−101^
STAT2	Activation	0.44, *p* = 1.6 × 10^−45^	0.45, *p* = 1.5 × 10^−26^
STAT3	Activation	0.11, *p* = 1.0 × 10^−2^	0.26, *p* = 2.3 × 10^−9^
STAT4	Unknown	0.47, *p* = 9.8 × 10^−31^	0.55, *p* = 5.8 × 10^−41^

**Table 4:** Predicted miRNA–transcription factor pairs that may indirectly regulate *IRF1*.

miRNA	Predicted Target	Correlation with Target Gene in LUAD	Correlation with Target Gene in LUSC
hsa-miR-183	NFKB1	−0.23, *p* = 7.9 × 10^−5^	−0.32, *p* = 1.7 × 10^−9^
hsa-miR-141	STAT4	−0.20, *p* = 1.1 × 10^−3^	−0.42, *p* = 1.0 × 10^−35^

## Data Availability

The original contributions presented in this study are included in the article and Supplementary Materials. Further inquiries can be directed to the corresponding author.

## Supplementary Materials

The following supporting information can be downloaded at: https://www.mdpi.com/article/10.3390/ijms27125192/s1.
