# Supplementary material for: A Multi-Level miRNA Regulatory Network Associated with IRF1 Expression in Non-Small Cell Lung Cancer: In Silico Identification of Candidate Biomarkers for Immunotherapy Response
Source: Int J Mol Sci. 2026 Jun 8;27(12):5192. doi: 10.3390/ijms27125192 (PMC13300628; doi:10.3390/ijms27125192)
Supplement: Supplementary file 1 [file ijms-27-05192-s001.zip › ijms-4286133-supplementary/Supplementary Figure S1.pptx]

## Slide 1
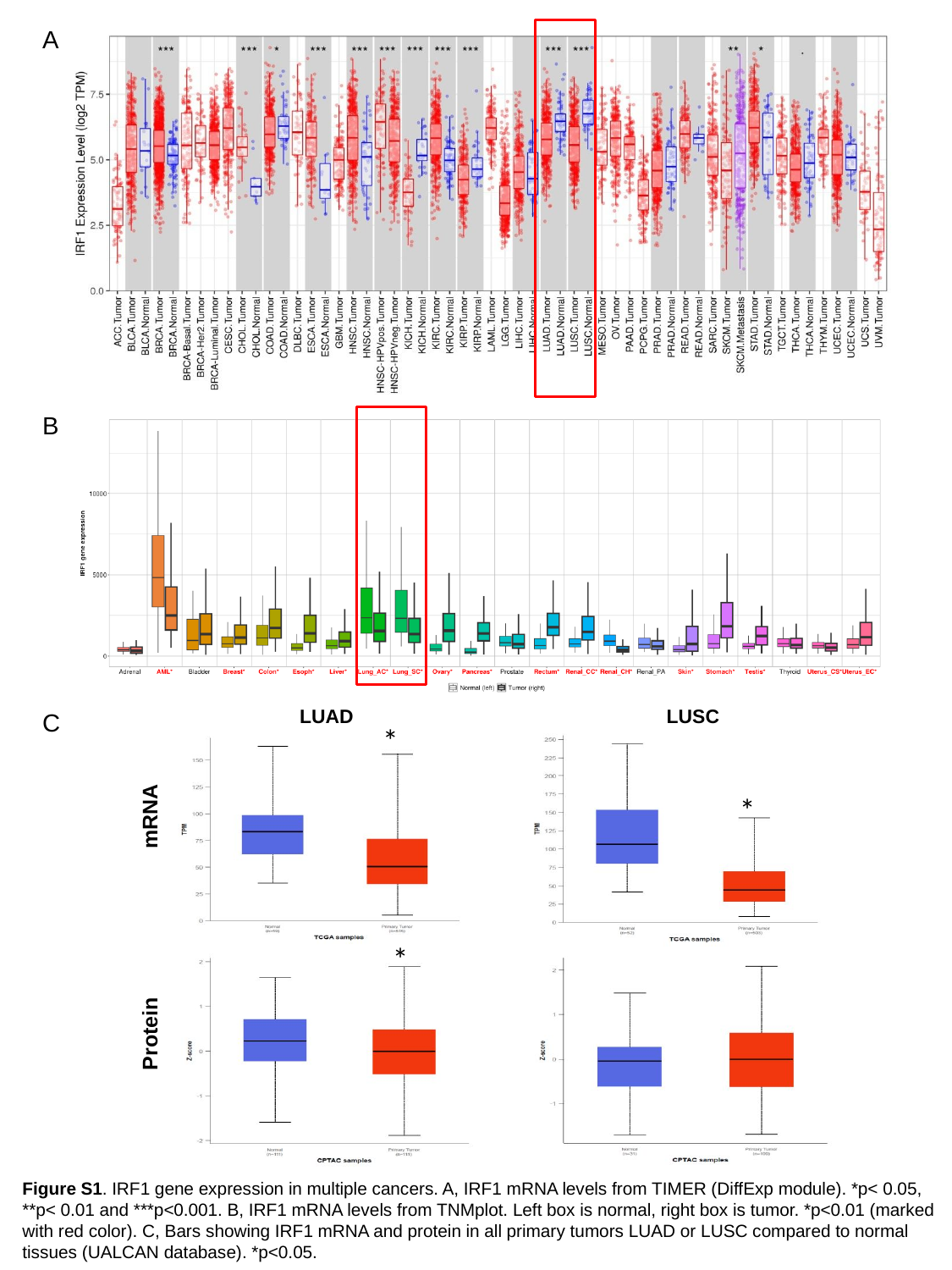

A
B
LUAD
LUSC
C
*
*
Protein mRNA
*
Figure S1. IRF1 gene expression in multiple cancers. A, IRF1 mRNA levels from TIMER (DiffExp module). *p< 0.05, **p< 0.01 and ***p<0.001. B, IRF1 mRNA levels from TNMplot. Left box is normal, right box is tumor. *p<0.01 (marked with red color). C, Bars showing IRF1 mRNA and protein in all primary tumors LUAD or LUSC compared to normal tissues (UALCAN database). *p<0.05.
