# Supplementary material for: A Multi-Level miRNA Regulatory Network Associated with IRF1 Expression in Non-Small Cell Lung Cancer: In Silico Identification of Candidate Biomarkers for Immunotherapy Response
Source: Int J Mol Sci. 2026 Jun 8;27(12):5192. doi: 10.3390/ijms27125192 (PMC13300628; doi:10.3390/ijms27125192)
Supplement: Supplementary file 1 [file ijms-27-05192-s001.zip › ijms-4286133-supplementary/Supplementary Figure S2.pptx]

## Slide 1
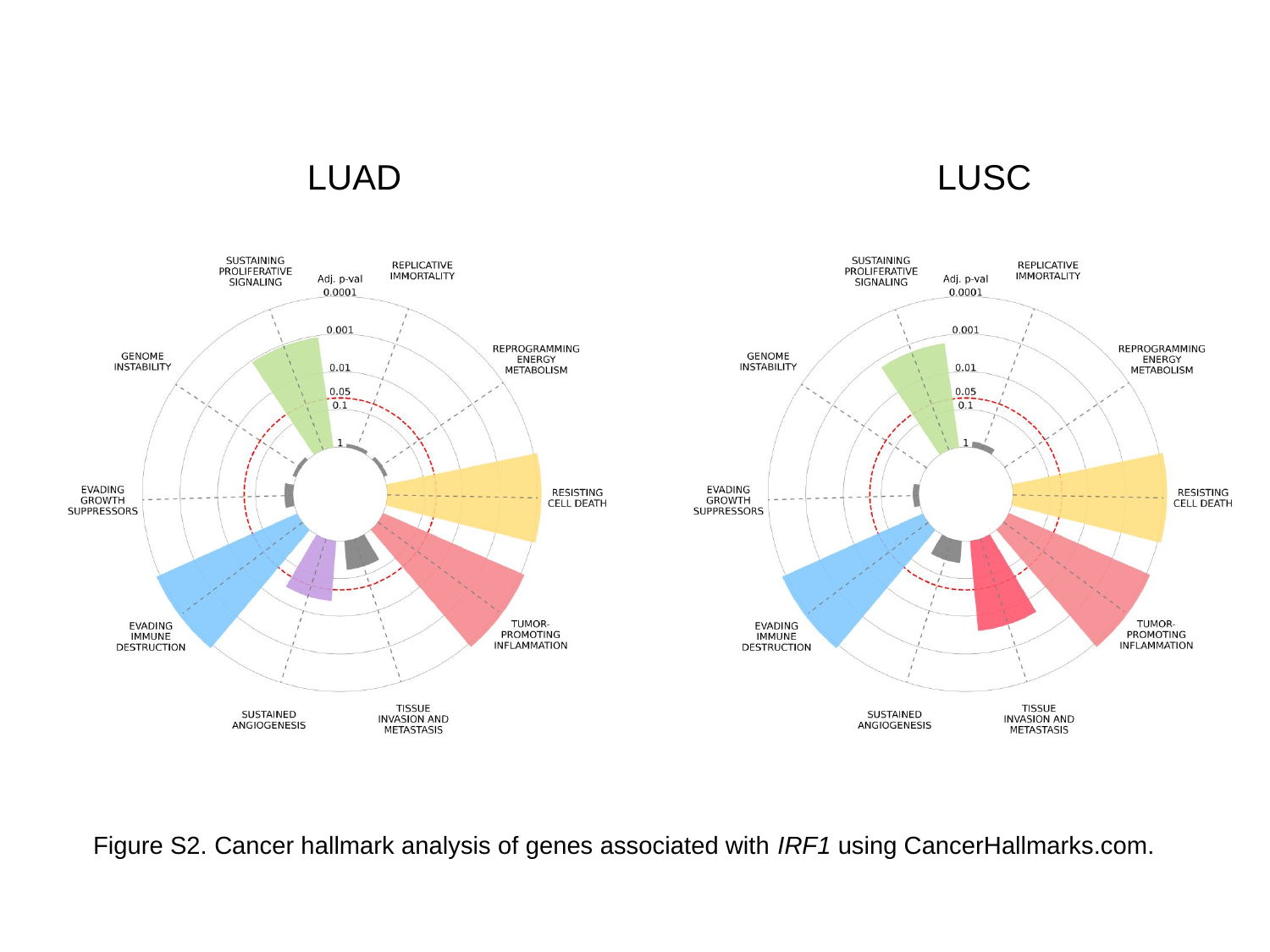

LUAD LUSC
Figure S2. Cancer hallmark analysis of genes associated with IRF1 using CancerHallmarks.com.
