# Supplementary material for: A Multi-Level miRNA Regulatory Network Associated with IRF1 Expression in Non-Small Cell Lung Cancer: In Silico Identification of Candidate Biomarkers for Immunotherapy Response
Source: Int J Mol Sci. 2026 Jun 8;27(12):5192. doi: 10.3390/ijms27125192 (PMC13300628; doi:10.3390/ijms27125192)
Supplement: Supplementary file 1 [file ijms-27-05192-s001.zip › ijms-4286133-supplementary/Supplementary Figure S3.pptx]

## Slide 1
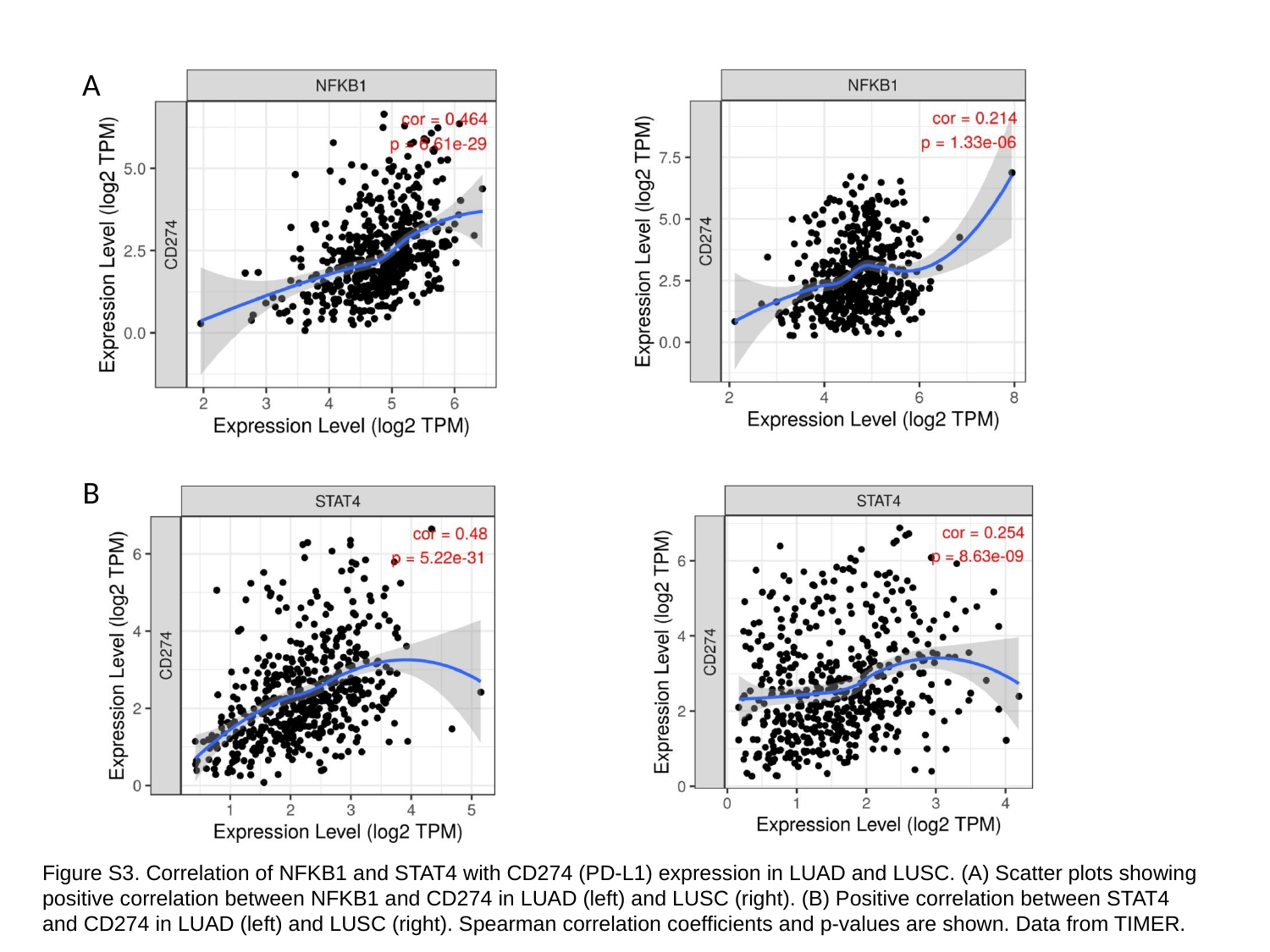

A
B
Figure S3. Correlation of NFKB1 and STAT4 with CD274 (PD‑L1) expression in LUAD and LUSC. (A) Scatter plots showing positive correlation between NFKB1 and CD274 in LUAD (left) and LUSC (right). (B) Positive correlation between STAT4 and CD274 in LUAD (left) and LUSC (right). Spearman correlation coefficients and p‑values are shown. Data from TIMER.
