# Supplementary material for: A Multi-Level miRNA Regulatory Network Associated with IRF1 Expression in Non-Small Cell Lung Cancer: In Silico Identification of Candidate Biomarkers for Immunotherapy Response
Source: Int J Mol Sci. 2026 Jun 8;27(12):5192. doi: 10.3390/ijms27125192 (PMC13300628; doi:10.3390/ijms27125192)
Supplement: Supplementary file 1 [file ijms-27-05192-s001.zip › ijms-4286133-supplementary/Supplementary Table S1.pdf]

Supplementary Table S1. List of genes with the highest correlation coefficient with the IRF1 gene level in LUAD samples.

Target Gene/Attribute	Spearman Correlation	P-value	FDR (BH)	Event_SD	Event_TD
IRF1	1.000e+00	1.000e-71	1.000e-69	3.2e+02	3.20e+02
GBP4	8.371e-01	1.000e-71	1.000e-69	3.2e+02	3.20e+02
GBP1	8.346e-01	1.000e-71	1.000e-69	3.2e+02	3.20e+02
PSMB9	8.292e-01	1.000e-71	1.000e-69	3.2e+02	3.20e+02
NKG7	8.146e-01	1.000e-71	1.000e-69	3.2e+02	3.20e+02
TAP1	8.046e-01	1.000e-71	1.000e-69	3.2e+02	3.20e+02
CCL5	7.991e-01	1.000e-71	1.000e-69	3.2e+02	3.20e+02
GZMA	7.859e-01	1.000e-71	1.000e-69	3.2e+02	3.20e+02
FASLG	7.850e-01	1.000e-71	1.000e-69	3.2e+02	3.19e+02
IL15RA	7.767e-01	1.000e-71	1.000e-69	3.2e+02	3.20e+02
WARS	7.759e-01	1.000e-71	1.000e-69	3.2e+02	3.20e+02
GBP5	7.736e-01	1.000e-71	1.000e-69	3.2e+02	3.20e+02
APOL3	7.704e-01	1.000e-71	1.000e-69	3.2e+02	3.20e+02
CXCL10	7.562e-01	1.000e-71	1.000e-69	3.2e+02	3.20e+02
GZMH	7.556e-01	1.000e-71	1.000e-69	3.2e+02	3.19e+02
GZMB	7.552e-01	1.000e-71	1.000e-69	3.2e+02	3.20e+02
IL18BP	7.519e-01	1.000e-71	1.000e-69	3.2e+02	3.20e+02
CD8A	7.507e-01	1.000e-71	1.000e-69	3.2e+02	3.20e+02
TAP2	7.495e-01	1.000e-71	1.000e-69	3.2e+02	3.20e+02
CXCL9	7.431e-01	1.000e-71	1.000e-69	3.2e+02	3.20e+02
UBE2L6	7.401e-01	1.000e-71	1.000e-69	3.2e+02	3.20e+02
KLRK1	7.394e-01	1.000e-71	1.000e-69	3.2e+02	3.20e+02
SIRPG	7.394e-01	1.000e-71	1.000e-69	3.2e+02	3.20e+02
CXCL11	7.382e-01	1.000e-71	1.000e-69	3.2e+02	3.20e+02
SLA2	7.368e-01	1.000e-71	1.000e-69	3.2e+02	3.20e+02
IL12RB1	7.333e-01	1.000e-71	1.000e-69	3.2e+02	3.20e+02
CCL4	7.332e-01	1.000e-71	1.000e-69	3.2e+02	3.20e+02
ETV7	7.329e-01	1.000e-71	1.000e-69	3.2e+02	3.20e+02
STAT1	7.328e-01	1.000e-71	1.000e-69	3.2e+02	3.20e+02
C5orf56	7.325e-01	1.000e-71	1.000e-69	3.2e+02	3.20e+02
IL2RB	7.299e-01	1.000e-71	1.000e-69	3.2e+02	3.20e+02
NLRC5	7.296e-01	1.000e-71	1.000e-69	3.2e+02	3.20e+02
ZNF683	7.256e-01	1.000e-71	1.000e-69	3.2e+02	3.20e+02
BATF2	7.237e-01	1.000e-71	1.000e-69	3.2e+02	3.20e+02
CD247	7.205e-01	1.000e-71	1.000e-69	3.2e+02	3.20e+02
FAM26F	7.167e-01	1.000e-71	1.000e-69	3.2e+02	3.20e+02
UBD	7.161e-01	1.000e-71	1.000e-69	3.2e+02	3.20e+02
PRF1	7.108e-01	1.000e-71	1.000e-69	3.2e+02	3.20e+02
LAG3	7.085e-01	1.000e-71	1.000e-69	3.2e+02	3.20e+02
KLRD1	7.077e-01	1.000e-71	1.000e-69	3.2e+02	3.20e+02
TIGIT	7.073e-01	1.000e-71	1.000e-69	3.2e+02	3.20e+02
PTPN7	7.067e-01	1.000e-71	1.000e-69	3.2e+02	3.20e+02
CXCR6	7.032e-01	1.000e-71	1.000e-69	3.2e+02	3.20e+02
CD3E	7.016e-01	1.000e-71	1.000e-69	3.2e+02	3.20e+02
HLA-F	7.010e-01	1.000e-71	1.000e-69	3.2e+02	3.20e+02
PSMB10	6.995e-01	1.000e-71	1.000e-69	3.2e+02	3.20e+02
TBX21	6.978e-01	1.000e-71	1.000e-69	3.2e+02	3.20e+02
CCR5	6.959e-01	1.000e-71	1.000e-69	3.2e+02	3.20e+02
IL15	6.931e-01	1.000e-71	1.000e-69	3.2e+02	3.20e+02
GBP2	6.922e-01	1.000e-71	1.000e-69	3.2e+02	3.20e+02
GNLY	6.916e-01	1.000e-71	1.000e-69	3.2e+02	3.20e+02
PDCD1	6.914e-01	1.000e-71	1.000e-69	3.2e+02	3.20e+02
CD3D	6.909e-01	1.000e-71	1.000e-69	3.2e+02	3.20e+02

Target Gene/Attribute	Spearman Correlation	P-value	FDR (BH)	Event_SD	Event_TD
HLA-B	6.869e-01	1.000e-71	1.000e-69	3.2e+02	3.20e+02
APOL6	6.828e-01	1.000e-71	1.000e-69	3.2e+02	3.20e+02
SAMD9L	6.818e-01	1.000e-71	1.000e-69	3.2e+02	3.20e+02
LCK	6.809e-01	1.000e-71	1.000e-69	3.2e+02	3.20e+02
HAPLN3	6.783e-01	1.000e-71	1.000e-69	3.2e+02	3.20e+02
PDCD1LG2	6.751e-01	1.000e-71	1.000e-69	3.2e+02	3.20e+02
IL2RG	6.734e-01	1.000e-71	1.000e-69	3.2e+02	3.20e+02
PSME2	6.726e-01	1.000e-71	1.000e-69	3.2e+02	3.20e+02
CD6	6.686e-01	1.000e-71	1.000e-69	3.2e+02	3.20e+02
PYHIN1	6.669e-01	1.000e-71	1.000e-69	3.2e+02	3.19e+02
CD8B	6.640e-01	1.000e-71	1.000e-69	3.2e+02	3.20e+02
ICOS	6.615e-01	1.000e-71	1.000e-69	3.2e+02	3.20e+02
IL18RAP	6.615e-01	1.000e-71	1.000e-69	3.2e+02	3.20e+02
PSMB8	6.615e-01	1.000e-71	1.000e-69	3.2e+02	3.20e+02
PTPRCAP	6.615e-01	1.000e-71	1.000e-69	3.2e+02	3.20e+02
B2M	6.611e-01	1.000e-71	1.000e-69	3.2e+02	3.20e+02
PSTPIP1	6.598e-01	1.000e-71	1.000e-69	3.2e+02	3.20e+02
APOBEC3G	6.593e-01	1.000e-71	1.000e-69	3.2e+02	3.20e+02
SIT1	6.568e-01	1.000e-71	1.000e-69	3.2e+02	3.19e+02
SH2D1A	6.558e-01	1.000e-71	1.000e-69	3.2e+02	3.19e+02
GIMAP5	6.550e-01	1.000e-71	1.000e-69	3.2e+02	3.20e+02
IL4I1	6.549e-01	1.000e-71	1.000e-69	3.2e+02	3.20e+02
IL21R	6.500e-01	1.000e-71	1.000e-69	3.2e+02	3.20e+02
LTA	6.500e-01	1.000e-71	1.000e-69	3.2e+02	3.19e+02
UBASH3A	6.500e-01	1.000e-71	1.000e-69	3.2e+02	3.20e+02
ACAP1	6.493e-01	1.000e-71	1.000e-69	3.2e+02	3.20e+02
TBC1D10C	6.493e-01	1.000e-71	1.000e-69	3.2e+02	3.20e+02
ARHGAP9	6.461e-01	1.000e-71	1.000e-69	3.2e+02	3.20e+02
CD72	6.444e-01	1.000e-71	1.000e-69	3.2e+02	3.20e+02
IDO1	6.443e-01	1.000e-71	1.000e-69	3.2e+02	3.20e+02
THEMIS	6.432e-01	1.000e-71	1.000e-69	3.2e+02	3.20e+02
BTN3A3	6.423e-01	1.000e-71	1.000e-69	3.2e+02	3.20e+02
HCST	6.414e-01	1.000e-71	1.000e-69	3.2e+02	3.20e+02
NCF1	6.412e-01	1.000e-71	1.000e-69	3.2e+02	3.20e+02
CD274	6.409e-01	1.000e-71	1.000e-69	3.2e+02	3.20e+02
MIR155HG	6.406e-01	1.000e-71	1.000e-69	3.2e+02	3.20e+02
CXCR3	6.386e-01	1.000e-71	1.000e-69	3.2e+02	3.20e+02
SAMD3	6.378e-01	1.000e-71	1.000e-69	3.2e+02	3.20e+02
HLA-E	6.375e-01	1.000e-71	1.000e-69	3.2e+02	3.20e+02
LILRB2	6.374e-01	1.000e-71	1.000e-69	3.2e+02	3.20e+02
SLAMF8	6.372e-01	1.000e-71	1.000e-69	3.2e+02	3.20e+02
HCP5	6.348e-01	1.000e-71	1.000e-69	3.2e+02	3.20e+02
CYTH4	6.347e-01	1.000e-71	1.000e-69	3.2e+02	3.20e+02
CD244	6.342e-01	1.000e-71	1.000e-69	3.2e+02	3.20e+02
ZAP70	6.332e-01	1.000e-71	1.000e-69	3.2e+02	3.20e+02
LCP2	6.324e-01	1.000e-71	1.000e-69	3.2e+02	3.20e+02
EPSTI1	6.311e-01	1.000e-71	1.000e-69	3.2e+02	3.20e+02
BTN3A1	6.296e-01	1.000e-71	1.000e-69	3.2e+02	3.20e+02
CD96	6.278e-01	1.000e-71	1.000e-69	3.2e+02	3.20e+02
ITGB7	6.258e-01	1.000e-71	1.000e-69	3.2e+02	3.20e+02
RASAL3	6.258e-01	1.000e-71	1.000e-69	3.2e+02	3.20e+02
LILRB1	6.250e-01	1.000e-71	1.000e-69	3.2e+02	3.20e+02
FYB	6.230e-01	1.000e-71	1.000e-69	3.2e+02	3.20e+02
TNFSF13B	6.208e-01	1.000e-71	1.000e-69	3.2e+02	3.20e+02
CD7	6.203e-01	1.000e-71	1.000e-69	3.2e+02	3.20e+02
GFI1	6.195e-01	1.000e-71	1.000e-69	3.2e+02	3.20e+02

Target Gene/Attribute	Spearman Correlation	P-value	FDR (BH)	Event_SD	Event_TD
ITGAL	6.191e-01	1.000e-71	1.000e-69	3.2e+02	3.20e+02
SLFN12L	6.176e-01	1.000e-71	1.000e-69	3.2e+02	3.20e+02
ZBED2	6.176e-01	1.000e-71	1.000e-69	3.2e+02	3.20e+02
LILRB4	6.167e-01	1.000e-71	1.000e-69	3.2e+02	3.20e+02
CCL4L2	6.146e-01	1.000e-71	1.000e-69	3.2e+02	3.20e+02
CTLA4	6.145e-01	1.000e-71	1.000e-69	3.2e+02	3.20e+02
TMEM140	6.137e-01	1.000e-71	1.000e-69	3.2e+02	3.20e+02
FCGR1B	6.134e-01	1.000e-71	1.000e-69	3.2e+02	3.20e+02
C1QA	6.128e-01	1.000e-71	1.000e-69	3.2e+02	3.20e+02
WAS	6.127e-01	1.000e-71	1.000e-69	3.2e+02	3.20e+02
ASB2	6.118e-01	1.000e-71	1.000e-69	3.2e+02	3.20e+02
GIMAP4	6.105e-01	1.000e-71	1.000e-69	3.2e+02	3.20e+02
LAP3	6.102e-01	1.000e-71	1.000e-69	3.2e+02	3.20e+02
PLEK	6.102e-01	1.000e-71	1.000e-69	3.2e+02	3.20e+02
RTP4	6.089e-01	1.000e-71	1.000e-69	3.2e+02	3.20e+02
ABCD2	6.077e-01	1.000e-71	1.000e-69	3.2e+02	3.20e+02
SASH3	6.073e-01	1.000e-71	1.000e-69	3.2e+02	3.20e+02
MMP25	6.068e-01	1.000e-71	1.000e-69	3.2e+02	3.20e+02
SEPT1	6.045e-01	1.000e-71	1.000e-69	3.2e+02	3.20e+02
ZBP1	6.045e-01	1.000e-71	1.000e-69	3.2e+02	3.20e+02
SIGLEC10	6.043e-01	1.000e-71	1.000e-69	3.2e+02	3.20e+02
AOAH	6.042e-01	1.000e-71	1.000e-69	3.2e+02	3.20e+02
EOMES	6.035e-01	1.000e-71	1.000e-69	3.2e+02	3.20e+02
FERMT3	6.016e-01	1.000e-71	1.000e-69	3.2e+02	3.20e+02
GZMK	6.008e-01	1.000e-71	1.000e-69	3.2e+02	3.20e+02
KCNJ10	6.002e-01	1.000e-71	1.000e-69	3.2e+02	3.19e+02
SLAMF6	5.997e-01	1.000e-71	1.000e-69	3.2e+02	3.20e+02
ABI3	5.985e-01	1.000e-71	1.000e-69	3.2e+02	3.20e+02
ADAMDEC1	5.985e-01	1.000e-71	1.000e-69	3.2e+02	3.19e+02
SLAMF1	5.966e-01	1.000e-71	1.000e-69	3.2e+02	3.20e+02
SP140	5.955e-01	1.000e-71	1.000e-69	3.2e+02	3.20e+02
EBI3	5.951e-01	1.000e-71	1.000e-69	3.2e+02	3.19e+02
SLC15A3	5.945e-01	1.000e-71	1.000e-69	3.2e+02	3.20e+02
FCGR1C	5.943e-01	1.000e-71	1.000e-69	3.2e+02	3.20e+02
MAP4K1	5.918e-01	1.000e-71	1.000e-69	3.2e+02	3.20e+02
NLRC3	5.917e-01	1.000e-71	1.000e-69	3.2e+02	3.20e+02
CARD16	5.904e-01	1.000e-71	1.000e-69	3.2e+02	3.20e+02
CASP1	5.903e-01	1.000e-71	1.000e-69	3.2e+02	3.20e+02
TRAF1	5.898e-01	1.000e-71	1.000e-69	3.2e+02	3.20e+02
HLA-C	5.897e-01	1.000e-71	1.000e-69	3.2e+02	3.20e+02
CD3G	5.871e-01	1.000e-71	1.000e-69	3.2e+02	3.19e+02
FCGR1A	5.865e-01	1.000e-71	1.000e-69	3.2e+02	3.20e+02
STX11	5.857e-01	1.000e-71	1.000e-69	3.2e+02	3.20e+02
BTN3A2	5.853e-01	1.000e-71	1.000e-69	3.2e+02	3.20e+02
JAK2	5.842e-01	1.000e-71	1.000e-69	3.2e+02	3.20e+02
GRIN3A	5.839e-01	1.000e-71	1.000e-69	3.2e+02	3.20e+02
C1QB	5.834e-01	1.000e-71	1.000e-69	3.2e+02	3.20e+02
CD53	5.833e-01	1.000e-71	1.000e-69	3.2e+02	3.20e+02
GPR65	5.833e-01	1.000e-71	1.000e-69	3.2e+02	3.20e+02
ITK	5.829e-01	1.000e-71	1.000e-69	3.2e+02	3.20e+02
AIM2	5.827e-01	1.000e-71	1.000e-69	3.2e+02	3.20e+02
SNX20	5.825e-01	1.000e-71	1.000e-69	3.2e+02	3.20e+02
IFI30	5.819e-01	1.000e-71	1.000e-69	3.2e+02	3.20e+02
SLAMF7	5.808e-01	1.000e-71	1.000e-69	3.2e+02	3.20e+02
TRIM21	5.803e-01	1.000e-71	1.000e-69	3.2e+02	3.20e+02
PARP12	5.802e-01	1.000e-71	1.000e-69	3.2e+02	3.20e+02

Target Gene/Attribute	Spearman Correlation	P-value	FDR (BH)	Event_SD	Event_TD
C1QC	5.799e-01	1.000e-71	1.000e-69	3.2e+02	3.20e+02
CORO1A	5.787e-01	1.000e-71	1.000e-69	3.2e+02	3.20e+02
SOD2	5.786e-01	1.000e-71	1.000e-69	3.2e+02	3.20e+02
APOL2	5.779e-01	1.000e-71	1.000e-69	3.2e+02	3.20e+02
GJD3	5.770e-01	1.000e-71	1.000e-69	3.2e+02	3.20e+02
SAMSN1	5.768e-01	1.000e-71	1.000e-69	3.2e+02	3.20e+02
HLA-A	5.766e-01	1.000e-71	1.000e-69	3.2e+02	3.20e+02
GIMAP2	5.757e-01	1.000e-71	1.000e-69	3.2e+02	3.20e+02
GPR84	5.753e-01	1.000e-71	1.000e-69	3.2e+02	3.19e+02
SLC31A2	5.750e-01	1.000e-71	1.000e-69	3.2e+02	3.20e+02
RARRES3	5.741e-01	1.000e-71	1.000e-69	3.2e+02	3.20e+02
BIN2	5.740e-01	1.000e-71	1.000e-69	3.2e+02	3.20e+02
FCER1G	5.736e-01	1.000e-71	1.000e-69	3.2e+02	3.20e+02
PTPRC	5.732e-01	1.000e-71	1.000e-69	3.2e+02	3.20e+02
XAF1	5.732e-01	1.000e-71	1.000e-69	3.2e+02	3.20e+02
FAM78A	5.730e-01	1.000e-71	1.000e-69	3.2e+02	3.20e+02
IL10RA	5.730e-01	1.000e-71	1.000e-69	3.2e+02	3.20e+02
NUB1	5.716e-01	1.000e-71	1.000e-69	3.2e+02	3.20e+02
GIMAP7	5.711e-01	1.000e-71	1.000e-69	3.2e+02	3.20e+02
RAC2	5.708e-01	1.000e-71	1.000e-69	3.2e+02	3.20e+02
JAK3	5.706e-01	1.000e-71	1.000e-69	3.2e+02	3.20e+02
KLHDC7B	5.698e-01	1.000e-71	1.000e-69	3.2e+02	3.20e+02
LAT	5.688e-01	1.000e-71	1.000e-69	3.2e+02	3.20e+02
CD5	5.687e-01	1.000e-71	1.000e-69	3.2e+02	3.20e+02
LPXN	5.669e-01	1.000e-71	1.000e-69	3.2e+02	3.20e+02
TFEC	5.656e-01	1.000e-71	1.000e-69	3.2e+02	3.20e+02
CD48	5.654e-01	1.000e-71	1.000e-69	3.2e+02	3.20e+02
PVRIG	5.647e-01	1.000e-71	1.000e-69	3.2e+02	3.20e+02
CCL3	5.634e-01	1.000e-71	1.000e-69	3.2e+02	3.20e+02
SH2D2A	5.634e-01	1.000e-71	1.000e-69	3.2e+02	3.20e+02
TMEM229B	5.632e-01	1.000e-71	1.000e-69	3.2e+02	3.20e+02
CCL8	5.625e-01	1.000e-71	1.000e-69	3.2e+02	3.20e+02
GPR114	5.618e-01	1.000e-71	1.000e-69	3.2e+02	3.20e+02
IFI35	5.587e-01	1.000e-71	1.000e-69	3.2e+02	3.20e+02
HAVCR2	5.584e-01	1.000e-71	1.000e-69	3.2e+02	3.20e+02
MYO1F	5.565e-01	1.000e-71	1.000e-69	3.2e+02	3.20e+02
CD40	5.542e-01	1.000e-71	1.000e-69	3.2e+02	3.20e+02
NCF1B	5.539e-01	1.000e-71	1.000e-69	3.2e+02	3.20e+02
CLEC4E	5.537e-01	1.000e-71	1.000e-69	3.2e+02	3.19e+02
HCG26	5.522e-01	1.000e-71	1.000e-69	3.2e+02	3.20e+02
HLA-H	5.521e-01	1.000e-71	1.000e-69	3.2e+02	3.20e+02
BCL2A1	5.514e-01	1.000e-71	1.000e-69	3.2e+02	3.20e+02
CST7	5.506e-01	1.000e-71	1.000e-69	3.2e+02	3.20e+02
CSF2RB	5.491e-01	1.000e-71	1.000e-69	3.2e+02	3.20e+02
WIPF1	5.484e-01	1.000e-71	1.000e-69	3.2e+02	3.20e+02
ARHGAP25	5.478e-01	1.000e-71	1.000e-69	3.2e+02	3.20e+02
SELPLG	5.450e-01	1.000e-71	1.000e-69	3.2e+02	3.20e+02
FOXP3	5.429e-01	1.000e-71	1.000e-69	3.2e+02	3.20e+02
C19orf38	5.426e-01	1.000e-71	1.000e-69	3.2e+02	3.20e+02
APBB1IP	5.418e-01	1.000e-71	1.000e-69	3.2e+02	3.20e+02
TNFRSF1B	5.415e-01	1.000e-71	1.000e-69	3.2e+02	3.20e+02
FCGR3A	5.410e-01	1.000e-71	1.000e-69	3.2e+02	3.20e+02
ARHGAP30	5.403e-01	1.000e-71	1.000e-69	3.2e+02	3.20e+02
CIITA	5.386e-01	1.000e-71	1.000e-69	3.2e+02	3.20e+02
OPTN	5.382e-01	1.000e-71	1.000e-69	3.2e+02	3.20e+02
IKZF1	5.366e-01	1.000e-71	1.000e-69	3.2e+02	3.20e+02

Target Gene/Attribute	Spearman Correlation	P-value	FDR (BH)	Event_SD	Event_TD
TNFAIP3	5.355e-01	1.000e-71	1.000e-69	3.2e+02	3.20e+02
AIF1	5.349e-01	1.000e-71	1.000e-69	3.2e+02	3.20e+02
LAPTM5	5.348e-01	1.000e-71	1.000e-69	3.2e+02	3.20e+02
PLEKHO1	5.348e-01	1.000e-71	1.000e-69	3.2e+02	3.20e+02
GVIN1	5.344e-01	1.000e-71	1.000e-69	3.2e+02	3.20e+02
IL32	5.337e-01	1.000e-71	1.000e-69	3.2e+02	3.20e+02
CD86	5.336e-01	1.000e-71	1.000e-69	3.2e+02	3.20e+02
GIMAP1	5.333e-01	1.000e-71	1.000e-69	3.2e+02	3.20e+02
GRAP2	5.332e-01	1.000e-71	1.000e-69	3.2e+02	3.20e+02
PIK3AP1	5.313e-01	1.000e-71	1.000e-69	3.2e+02	3.20e+02
NMI	5.306e-01	1.000e-71	1.000e-69	3.2e+02	3.20e+02
FGL2	5.303e-01	1.000e-71	1.000e-69	3.2e+02	3.20e+02
GMFG	5.301e-01	1.000e-71	1.000e-69	3.2e+02	3.20e+02
APOL1	5.297e-01	1.000e-71	1.000e-69	3.2e+02	3.20e+02
TAGAP	5.296e-01	1.000e-71	1.000e-69	3.2e+02	3.20e+02
AGAP2	5.295e-01	1.000e-71	1.000e-69	3.2e+02	3.20e+02
PARP9	5.293e-01	1.000e-71	1.000e-69	3.2e+02	3.20e+02
SLC2A6	5.285e-01	1.000e-71	1.000e-69	3.2e+02	3.20e+02
LAIR1	5.272e-01	1.000e-71	1.000e-69	3.2e+02	3.20e+02
GM2A	5.267e-01	1.000e-71	1.000e-69	3.2e+02	3.20e+02
IFIT3	5.264e-01	1.000e-71	1.000e-69	3.2e+02	3.20e+02
LOC100188949	5.249e-01	1.000e-71	1.000e-69	3.2e+02	3.20e+02
PML	5.247e-01	1.000e-71	1.000e-69	3.2e+02	3.20e+02
NCKAP1L	5.230e-01	1.000e-71	1.000e-69	3.2e+02	3.20e+02
CMKLR1	5.228e-01	1.000e-71	1.000e-69	3.2e+02	3.20e+02
GIMAP6	5.228e-01	1.000e-71	1.000e-69	3.2e+02	3.20e+02
LILRB3	5.218e-01	1.000e-71	1.000e-69	3.2e+02	3.20e+02
CD27	5.214e-01	1.000e-71	1.000e-69	3.2e+02	3.20e+02
MAFB	5.210e-01	1.000e-71	1.000e-69	3.2e+02	3.20e+02
TLR8	5.202e-01	1.000e-71	1.000e-69	3.2e+02	3.20e+02
CXCL13	5.194e-01	1.000e-71	1.000e-69	3.2e+02	3.20e+02
SERPINB9	5.192e-01	1.000e-71	1.000e-69	3.2e+02	3.20e+02
LOC606724	5.189e-01	1.000e-71	1.000e-69	3.2e+02	3.20e+02
LOC100233209	5.188e-01	1.000e-71	1.000e-69	3.2e+02	3.19e+02
FUT7	5.185e-01	1.000e-71	1.000e-69	3.2e+02	3.20e+02
P2RY10	5.185e-01	1.000e-71	1.000e-69	3.2e+02	3.20e+02
SCML4	5.173e-01	1.000e-71	1.000e-69	3.2e+02	3.19e+02
BTLA	5.172e-01	1.000e-71	1.000e-69	3.2e+02	3.20e+02
TMEM149	5.170e-01	1.000e-71	1.000e-69	3.2e+02	3.20e+02
PLEKHO2	5.169e-01	1.000e-71	1.000e-69	3.2e+02	3.20e+02
SLC7A7	5.168e-01	1.000e-71	1.000e-69	3.2e+02	3.20e+02
FMNL1	5.166e-01	1.000e-71	1.000e-69	3.2e+02	3.20e+02
PSME1	5.157e-01	1.000e-71	1.000e-69	3.2e+02	3.20e+02
FAM115C	5.153e-01	1.000e-71	1.000e-69	3.2e+02	3.20e+02
DOCK2	5.144e-01	1.000e-71	1.000e-69	3.2e+02	3.20e+02
PTGDR	5.144e-01	1.000e-71	1.000e-69	3.2e+02	3.19e+02
CD14	5.124e-01	1.000e-71	1.000e-69	3.2e+02	3.20e+02
TAPBP	5.115e-01	1.000e-71	1.000e-69	3.2e+02	3.20e+02
SECTM1	5.114e-01	1.000e-71	1.000e-69	3.2e+02	3.20e+02
TYMP	5.105e-01	1.000e-71	1.000e-69	3.2e+02	3.20e+02
TARP	5.104e-01	1.000e-71	1.000e-69	3.2e+02	3.19e+02
STAC3	5.101e-01	1.000e-71	1.000e-69	3.2e+02	3.20e+02
STAT5A	5.098e-01	1.000e-71	1.000e-69	3.2e+02	3.20e+02
ARHGAP15	5.086e-01	1.000e-71	1.000e-69	3.2e+02	3.20e+02
CYTIP	5.076e-01	1.000e-71	1.000e-69	3.2e+02	3.20e+02
EVI2B	5.070e-01	1.000e-71	1.000e-69	3.2e+02	3.20e+02

Target Gene/Attribute	Spearman Correlation	P-value	FDR (BH)	Event_SD	Event_TD
GNGT2	5.039e-01	1.000e-71	1.000e-69	3.2e+02	3.20e+02
P2RY13	5.033e-01	1.000e-71	1.000e-69	3.2e+02	3.20e+02
MICB	5.032e-01	1.000e-71	1.000e-69	3.2e+02	3.20e+02
SPI1	5.014e-01	1.000e-71	1.000e-69	3.2e+02	3.20e+02
PTPN22	5.010e-01	1.000e-71	1.000e-69	3.2e+02	3.20e+02
IL2RA	5.009e-01	1.000e-71	1.000e-69	3.2e+02	3.20e+02
