# Supplementary material for: A Multi-Level miRNA Regulatory Network Associated with IRF1 Expression in Non-Small Cell Lung Cancer: In Silico Identification of Candidate Biomarkers for Immunotherapy Response
Source: Int J Mol Sci. 2026 Jun 8;27(12):5192. doi: 10.3390/ijms27125192 (PMC13300628; doi:10.3390/ijms27125192)
Supplement: Supplementary file 1 [file ijms-27-05192-s001.zip › ijms-4286133-supplementary/Supplementary Table S2.pdf]

Supplementary Table S2. List of genes with the highest correlation coefficient with the IRF1 gene level in LUSC samples.

Target Gene/Attribute	Spearman Correlation	P-value	FDR (BH)	Event_SD	Event_TD
IRF1	1.000e+00	1.000e-143	1.000e-139	4.79e+02	4.79e+02
PSMB9	8.621e-01	6.808e-143	6.841e-139	4.79e+02	4.79e+02
APOL3	8.474e-01	2.995e-133	2.006e-129	4.79e+02	4.79e+02
GBP4	8.428e-01	1.905e-130	9.569e-127	4.79e+02	4.79e+02
TAP1	8.267e-01	3.131e-121	1.258e-117	4.79e+02	4.79e+02
NKG7	8.189e-01	4.258e-117	1.426e-113	4.79e+02	4.79e+02
GBP1	8.095e-01	2.176e-112	6.246e-109	4.79e+02	4.79e+02
HLA-B	7.989e-01	2.214e-107	5.561e-104	4.79e+02	4.79e+02
PSMB10	7.972e-01	1.331e-106	2.971e-103	4.79e+02	4.79e+02
APOL6	7.969e-01	1.827e-106	3.672e-103	4.79e+02	4.79e+02
FASLG	7.936e-01	5.594e-105	1.022e-101	4.79e+02	4.72e+02
HLA-F	7.928e-01	1.217e-104	2.038e-101	4.79e+02	4.79e+02
UBE2L6	7.886e-01	8.795e-103	1.360e-99	4.79e+02	4.79e+02
PRF1	7.876e-01	2.175e-102	3.122e-99	4.79e+02	4.79e+02
GZMH	7.852e-01	2.313e-101	3.098e-98	4.79e+02	4.78e+02
CCL4	7.845e-01	5.015e-101	6.299e-98	4.79e+02	4.79e+02
GZMA	7.794e-01	6.468e-99	7.646e-96	4.79e+02	4.78e+02
CXCR6	7.792e-01	7.970e-99	8.899e-96	4.79e+02	4.79e+02
SLA2	7.790e-01	9.376e-99	9.917e-96	4.79e+02	4.79e+02
CD2	7.785e-01	1.469e-98	1.476e-95	4.79e+02	4.79e+02
CD3D	7.766e-01	8.674e-98	8.301e-95	4.79e+02	4.79e+02
CD8A	7.755e-01	2.537e-97	2.318e-94	4.79e+02	4.79e+02
IL15RA	7.741e-01	9.113e-97	7.963e-94	4.79e+02	4.79e+02
C5orf56	7.738e-01	1.176e-96	9.846e-94	4.79e+02	4.79e+02
LAG3	7.730e-01	2.624e-96	2.109e-93	4.79e+02	4.79e+02
CD3E	7.657e-01	1.756e-93	1.357e-90	4.79e+02	4.79e+02
IFNG	7.656e-01	2.025e-93	1.508e-90	4.79e+02	4.48e+02
PSMB8	7.653e-01	2.482e-93	1.782e-90	4.79e+02	4.79e+02
B2M	7.652e-01	2.931e-93	2.031e-90	4.79e+02	4.79e+02
BATF2	7.648e-01	4.073e-93	2.728e-90	4.79e+02	4.79e+02
IL12RB1	7.635e-01	1.261e-92	8.174e-90	4.79e+02	4.79e+02
GZMB	7.623e-01	3.560e-92	2.236e-89	4.79e+02	4.79e+02
SIRPG	7.619e-01	5.157e-92	3.141e-89	4.79e+02	4.78e+02
CCL5	7.599e-01	2.781e-91	1.644e-88	4.79e+02	4.79e+02
CXCL10	7.594e-01	4.359e-91	2.503e-88	4.79e+02	4.79e+02
STAT1	7.501e-01	1.028e-87	5.739e-85	4.79e+02	4.79e+02
BTN3A3	7.480e-01	5.898e-87	3.204e-84	4.79e+02	4.79e+02
PDCD1	7.475e-01	8.546e-87	4.520e-84	4.79e+02	4.78e+02
CD7	7.463e-01	2.348e-86	1.210e-83	4.79e+02	4.79e+02
CD247	7.423e-01	5.541e-85	2.784e-82	4.79e+02	4.79e+02
UBASH3A	7.380e-01	1.651e-83	8.092e-81	4.79e+02	4.78e+02
CXCL9	7.379e-01	1.812e-83	8.670e-81	4.79e+02	4.79e+02
RARRES3	7.357e-01	9.891e-83	4.623e-80	4.79e+02	4.79e+02
KLRK1	7.353e-01	1.268e-82	5.792e-80	4.79e+02	4.79e+02
GBP5	7.352e-01	1.387e-82	6.192e-80	4.79e+02	4.79e+02
HLA-C	7.346e-01	2.148e-82	9.386e-80	4.79e+02	4.79e+02
UBA7	7.332e-01	6.531e-82	2.793e-79	4.79e+02	4.79e+02
CXCR3	7.325e-01	1.060e-81	4.439e-79	4.79e+02	4.79e+02
ETV7	7.321e-01	1.469e-81	6.024e-79	4.79e+02	4.79e+02
HLA-E	7.317e-01	2.028e-81	8.151e-79	4.79e+02	4.79e+02
CXCL11	7.306e-01	4.381e-81	1.726e-78	4.79e+02	4.79e+02
IL2RB	7.291e-01	1.419e-80	5.484e-78	4.79e+02	4.79e+02
SAMD9L	7.261e-01	1.303e-79	4.942e-77	4.79e+02	4.79e+02

Target Gene/Attribute	Spearman Correlation	P-value	FDR (BH)	Event_SD	Event_TD
EPSTI1	7.238e-01	6.853e-79	2.551e-76	4.79e+02	4.79e+02
HLA-A	7.193e-01	1.716e-77	6.269e-75	4.79e+02	4.79e+02
CD96	7.182e-01	3.854e-77	1.383e-74	4.79e+02	4.79e+02
CCR5	7.171e-01	8.540e-77	3.011e-74	4.79e+02	4.79e+02
NLRC5	7.170e-01	8.859e-77	3.070e-74	4.79e+02	4.79e+02
PTPN22	7.159e-01	1.958e-76	6.671e-74	4.79e+02	4.79e+02
CD74	7.155e-01	2.503e-76	8.383e-74	4.79e+02	4.79e+02
ICOS	7.152e-01	3.233e-76	1.065e-73	4.79e+02	4.78e+02
LAP3	7.141e-01	6.997e-76	2.268e-73	4.79e+02	4.79e+02
TIGIT	7.105e-01	7.967e-75	2.541e-72	4.79e+02	4.78e+02
BTN3A1	7.097e-01	1.415e-74	4.442e-72	4.79e+02	4.79e+02
TBC1D10C	7.072e-01	7.844e-74	2.425e-71	4.79e+02	4.79e+02
CST7	7.046e-01	4.334e-73	1.320e-70	4.79e+02	4.79e+02
CD3G	7.035e-01	9.283e-73	2.784e-70	4.79e+02	4.78e+02
HCST	7.034e-01	9.899e-73	2.926e-70	4.79e+02	4.79e+02
UBD	7.031e-01	1.185e-72	3.452e-70	4.79e+02	4.79e+02
IL2RG	7.005e-01	6.691e-72	1.921e-69	4.79e+02	4.79e+02
IL21R	7.004e-01	7.148e-72	2.023e-69	4.79e+02	4.79e+02
SLC15A3	7.000e-01	9.358e-72	2.612e-69	4.79e+02	4.79e+02
GIMAP5	6.995e-01	1.341e-71	3.693e-69	4.79e+02	4.79e+02
KLRD1	6.991e-01	1.711e-71	4.647e-69	4.79e+02	4.79e+02
TBX21	6.988e-01	2.049e-71	5.492e-69	4.79e+02	4.77e+02
CRTAM	6.963e-01	1.018e-70	2.692e-68	4.79e+02	4.78e+02
ZNF683	6.962e-01	1.089e-70	2.841e-68	4.79e+02	4.79e+02
SH2D1A	6.961e-01	1.181e-70	3.042e-68	4.79e+02	4.79e+02
TRIM22	6.959e-01	1.350e-70	3.434e-68	4.79e+02	4.79e+02
PYHIN1	6.956e-01	1.589e-70	3.991e-68	4.79e+02	4.77e+02
LCK	6.953e-01	1.927e-70	4.782e-68	4.79e+02	4.79e+02
PTPRCAP	6.949e-01	2.605e-70	6.385e-68	4.79e+02	4.79e+02
CTLA4	6.942e-01	4.112e-70	9.956e-68	4.79e+02	4.78e+02
GNLY	6.941e-01	4.219e-70	1.009e-67	4.79e+02	4.79e+02
PSME2	6.935e-01	6.391e-70	1.511e-67	4.79e+02	4.79e+02
IL15	6.925e-01	1.204e-69	2.788e-67	4.79e+02	4.79e+02
LCP2	6.925e-01	1.207e-69	2.788e-67	4.79e+02	4.79e+02
CD6	6.923e-01	1.373e-69	3.137e-67	4.79e+02	4.79e+02
CTSW	6.920e-01	1.613e-69	3.642e-67	4.79e+02	4.79e+02
ITGB7	6.916e-01	2.137e-69	4.771e-67	4.79e+02	4.79e+02
CIITA	6.912e-01	2.647e-69	5.847e-67	4.79e+02	4.79e+02
HLA-DRA	6.907e-01	3.662e-69	8.000e-67	4.79e+02	4.79e+02
CORO1A	6.903e-01	4.813e-69	1.040e-66	4.79e+02	4.79e+02
ABI3	6.890e-01	1.109e-68	2.370e-66	4.79e+02	4.79e+02
GBP2	6.888e-01	1.209e-68	2.557e-66	4.79e+02	4.79e+02
IL18BP	6.879e-01	2.181e-68	4.565e-66	4.79e+02	4.79e+02
CD244	6.842e-01	2.125e-67	4.403e-65	4.79e+02	4.79e+02
HLA-DMA	6.832e-01	4.055e-67	8.316e-65	4.79e+02	4.79e+02
HLA-DPA1	6.831e-01	4.300e-67	8.730e-65	4.79e+02	4.79e+02
GIMAP2	6.811e-01	1.461e-66	2.935e-64	4.79e+02	4.79e+02
SIT1	6.806e-01	1.936e-66	3.853e-64	4.79e+02	4.79e+02
TRAF3IP3	6.796e-01	3.475e-66	6.846e-64	4.79e+02	4.79e+02
ITK	6.786e-01	6.394e-66	1.248e-63	4.79e+02	4.79e+02
LTA	6.774e-01	1.294e-65	2.500e-63	4.79e+02	4.79e+02
SASH3	6.762e-01	2.796e-65	5.351e-63	4.79e+02	4.79e+02
GIMAP4	6.741e-01	9.378e-65	1.778e-62	4.79e+02	4.79e+02
GPR18	6.738e-01	1.112e-64	2.088e-62	4.79e+02	4.79e+02
FAM26F	6.737e-01	1.202e-64	2.236e-62	4.79e+02	4.79e+02
IL18RAP	6.729e-01	1.914e-64	3.529e-62	4.79e+02	4.78e+02

Target Gene/Attribute	Spearman Correlation	P-value	FDR (BH)	Event_SD	Event_TD
GNGT2	6.728e-01	1.992e-64	3.640e-62	4.79e+02	4.79e+02
HLA-DMB	6.728e-01	2.065e-64	3.739e-62	4.79e+02	4.79e+02
GPR174	6.724e-01	2.600e-64	4.665e-62	4.79e+02	4.64e+02
IDO1	6.724e-01	2.641e-64	4.697e-62	4.79e+02	4.79e+02
ARHGAP9	6.713e-01	4.867e-64	8.580e-62	4.79e+02	4.79e+02
BATF	6.703e-01	8.765e-64	1.532e-61	4.79e+02	4.79e+02
KLRC2	6.702e-01	9.309e-64	1.613e-61	4.79e+02	4.53e+02
ACAP1	6.678e-01	3.625e-63	6.227e-61	4.79e+02	4.79e+02
WARS	6.678e-01	3.715e-63	6.327e-61	4.79e+02	4.79e+02
JAKMIP1	6.674e-01	4.720e-63	7.926e-61	4.79e+02	4.75e+02
IFI30	6.674e-01	4.733e-63	7.926e-61	4.79e+02	4.79e+02
HAVCR2	6.668e-01	6.621e-63	1.100e-60	4.79e+02	4.79e+02
ITGAL	6.651e-01	1.745e-62	2.874e-60	4.79e+02	4.79e+02
HLA-H	6.644e-01	2.552e-62	4.170e-60	4.79e+02	4.79e+02
CXCR2P1	6.641e-01	3.119e-62	5.054e-60	4.79e+02	4.73e+02
TNFSF13B	6.641e-01	3.155e-62	5.073e-60	4.79e+02	4.79e+02
C1QA	6.634e-01	4.538e-62	7.239e-60	4.79e+02	4.79e+02
IL32	6.630e-01	5.934e-62	9.390e-60	4.79e+02	4.79e+02
CTSS	6.622e-01	9.303e-62	1.461e-59	4.79e+02	4.79e+02
BTN3A2	6.621e-01	9.468e-62	1.475e-59	4.79e+02	4.79e+02
TARP	6.604e-01	2.541e-61	3.928e-59	4.79e+02	4.74e+02
HLA-DPB1	6.600e-01	3.215e-61	4.932e-59	4.79e+02	4.79e+02
KLRC3	6.598e-01	3.430e-61	5.222e-59	4.79e+02	4.34e+02
GPR171	6.592e-01	4.877e-61	7.370e-59	4.79e+02	4.79e+02
CYTH4	6.584e-01	7.702e-61	1.155e-58	4.79e+02	4.79e+02
SEPT1	6.575e-01	1.269e-60	1.890e-58	4.79e+02	4.79e+02
PTPN7	6.572e-01	1.507e-60	2.227e-58	4.79e+02	4.79e+02
TNFAIP8L2	6.571e-01	1.579e-60	2.317e-58	4.79e+02	4.79e+02
PSME1	6.571e-01	1.598e-60	2.327e-58	4.79e+02	4.79e+02
TMEM229B	6.567e-01	1.915e-60	2.769e-58	4.79e+02	4.79e+02
TNFRSF9	6.557e-01	3.333e-60	4.785e-58	4.79e+02	4.78e+02
PTPRC	6.556e-01	3.661e-60	5.218e-58	4.79e+02	4.79e+02
TRAT1	6.552e-01	4.432e-60	6.272e-58	4.79e+02	4.74e+02
IFIT3	6.549e-01	5.340e-60	7.505e-58	4.79e+02	4.79e+02
FLT3LG	6.548e-01	5.630e-60	7.857e-58	4.79e+02	4.79e+02
TNFRSF1B	6.547e-01	5.890e-60	8.164e-58	4.79e+02	4.79e+02
GIMAP7	6.541e-01	8.323e-60	1.146e-57	4.79e+02	4.79e+02
SLAMF1	6.538e-01	9.630e-60	1.317e-57	4.79e+02	4.78e+02
ZBP1	6.538e-01	9.865e-60	1.340e-57	4.79e+02	4.78e+02
SP140	6.532e-01	1.362e-59	1.838e-57	4.79e+02	4.79e+02
GPR65	6.530e-01	1.512e-59	2.026e-57	4.79e+02	4.79e+02
TAPBPL	6.526e-01	1.850e-59	2.462e-57	4.79e+02	4.79e+02
GZMK	6.524e-01	2.065e-59	2.731e-57	4.79e+02	4.78e+02
SNX20	6.520e-01	2.584e-59	3.394e-57	4.79e+02	4.79e+02
HCP5	6.501e-01	7.313e-59	9.543e-57	4.79e+02	4.79e+02
SIGLEC10	6.484e-01	1.786e-58	2.315e-56	4.79e+02	4.79e+02
P2RY10	6.484e-01	1.808e-58	2.329e-56	4.79e+02	4.79e+02
ACSL5	6.481e-01	2.077e-58	2.659e-56	4.79e+02	4.79e+02
CD53	6.480e-01	2.170e-58	2.760e-56	4.79e+02	4.79e+02
WAS	6.477e-01	2.545e-58	3.216e-56	4.79e+02	4.79e+02
SLFN12L	6.477e-01	2.651e-58	3.330e-56	4.79e+02	4.79e+02
SCML4	6.474e-01	3.047e-58	3.803e-56	4.79e+02	4.78e+02
KLRC1	6.469e-01	3.929e-58	4.874e-56	4.79e+02	4.66e+02
CD48	6.467e-01	4.508e-58	5.559e-56	4.79e+02	4.79e+02
SLAMF8	6.458e-01	7.030e-58	8.607e-56	4.79e+02	4.79e+02
SLAMF6	6.458e-01	7.067e-58	8.607e-56	4.79e+02	4.79e+02

Target Gene/Attribute	Spearman Correlation	P-value	FDR (BH)	Event_SD	Event_TD
FCER1G	6.456e-01	8.118e-58	9.829e-56	4.79e+02	4.79e+02
TAP2	6.451e-01	1.050e-57	1.264e-55	4.79e+02	4.79e+02
NCF1	6.445e-01	1.398e-57	1.672e-55	4.79e+02	4.79e+02
LILRB2	6.442e-01	1.617e-57	1.923e-55	4.79e+02	4.79e+02
TMIGD2	6.430e-01	3.050e-57	3.606e-55	4.79e+02	4.60e+02
ARHGDIB	6.426e-01	3.942e-57	4.633e-55	4.79e+02	4.79e+02
FCGR1B	6.417e-01	6.296e-57	7.357e-55	4.79e+02	4.79e+02
NCF4	6.414e-01	7.181e-57	8.342e-55	4.79e+02	4.79e+02
CD80	6.410e-01	8.675e-57	1.002e-54	4.79e+02	4.79e+02
SAMSN1	6.406e-01	1.090e-56	1.252e-54	4.79e+02	4.79e+02
CD5	6.398e-01	1.650e-56	1.884e-54	4.79e+02	4.79e+02
FGL2	6.381e-01	3.930e-56	4.462e-54	4.79e+02	4.79e+02
C19orf38	6.366e-01	8.549e-56	9.638e-54	4.79e+02	4.79e+02
CD226	6.366e-01	8.584e-56	9.638e-54	4.79e+02	4.79e+02
SELPLG	6.351e-01	1.891e-55	2.112e-53	4.79e+02	4.79e+02
LAT2	6.348e-01	2.177e-55	2.417e-53	4.79e+02	4.79e+02
PARP14	6.346e-01	2.402e-55	2.653e-53	4.79e+02	4.79e+02
TRIM21	6.343e-01	2.789e-55	3.063e-53	4.79e+02	4.79e+02
IFI35	6.343e-01	2.820e-55	3.081e-53	4.79e+02	4.79e+02
LPXN	6.336e-01	3.982e-55	4.326e-53	4.79e+02	4.79e+02
FYB	6.334e-01	4.422e-55	4.777e-53	4.79e+02	4.79e+02
FCGR1A	6.332e-01	4.862e-55	5.225e-53	4.79e+02	4.79e+02
AIF1	6.327e-01	6.194e-55	6.621e-53	4.79e+02	4.79e+02
KIR2DL4	6.320e-01	8.980e-55	9.548e-53	4.79e+02	4.65e+02
LILRB1	6.318e-01	9.670e-55	1.023e-52	4.79e+02	4.79e+02
ZAP70	6.316e-01	1.084e-54	1.140e-52	4.79e+02	4.79e+02
C1QC	6.315e-01	1.143e-54	1.196e-52	4.79e+02	4.79e+02
NLRC3	6.305e-01	1.917e-54	1.996e-52	4.79e+02	4.79e+02
ABCD2	6.292e-01	3.565e-54	3.693e-52	4.79e+02	4.76e+02
GIMAP6	6.291e-01	3.893e-54	4.012e-52	4.79e+02	4.79e+02
GMFG	6.288e-01	4.493e-54	4.607e-52	4.79e+02	4.79e+02
C1QB	6.286e-01	4.824e-54	4.921e-52	4.79e+02	4.79e+02
VAMP5	6.276e-01	8.011e-54	8.132e-52	4.79e+02	4.79e+02
FGD2	6.271e-01	1.024e-53	1.034e-51	4.79e+02	4.79e+02
TAGAP	6.267e-01	1.228e-53	1.234e-51	4.79e+02	4.79e+02
DOK2	6.262e-01	1.605e-53	1.604e-51	4.79e+02	4.79e+02
HLA-DRB1	6.261e-01	1.684e-53	1.675e-51	4.79e+02	4.79e+02
NCKAP1L	6.250e-01	2.934e-53	2.894e-51	4.79e+02	4.79e+02
IL10RA	6.250e-01	2.938e-53	2.894e-51	4.79e+02	4.79e+02
KLRB1	6.244e-01	3.911e-53	3.834e-51	4.79e+02	4.75e+02
CD86	6.243e-01	4.072e-53	3.972e-51	4.79e+02	4.79e+02
XAF1	6.241e-01	4.562e-53	4.429e-51	4.79e+02	4.79e+02
ARHGAP15	6.234e-01	6.230e-53	6.020e-51	4.79e+02	4.79e+02
RHOH	6.231e-01	7.193e-53	6.917e-51	4.79e+02	4.79e+02
LST1	6.229e-01	8.204e-53	7.851e-51	4.79e+02	4.79e+02
IL4I1	6.225e-01	9.767e-53	9.303e-51	4.79e+02	4.79e+02
CCL4L2	6.223e-01	1.073e-52	1.017e-50	4.79e+02	4.79e+02
NCF1C	6.222e-01	1.110e-52	1.048e-50	4.79e+02	4.79e+02
BIN2	6.210e-01	2.013e-52	1.890e-50	4.79e+02	4.79e+02
GIMAP1	6.205e-01	2.550e-52	2.383e-50	4.79e+02	4.79e+02
FERMT3	6.195e-01	4.250e-52	3.954e-50	4.79e+02	4.79e+02
FCRL6	6.192e-01	4.878e-52	4.518e-50	4.79e+02	4.73e+02
IRF8	6.189e-01	5.518e-52	5.087e-50	4.79e+02	4.79e+02
BIRC3	6.187e-01	6.024e-52	5.528e-50	4.79e+02	4.79e+02
TIFAB	6.186e-01	6.575e-52	6.006e-50	4.79e+02	4.68e+02
RASAL3	6.182e-01	7.806e-52	7.098e-50	4.79e+02	4.79e+02

Target Gene/Attribute	Spearman Correlation	P-value	FDR (BH)	Event_SD	Event_TD
SPN	6.178e-01	9.484e-52	8.585e-50	4.79e+02	4.79e+02
SLC7A7	6.167e-01	1.598e-51	1.440e-49	4.79e+02	4.79e+02
LOC100188949	6.153e-01	3.105e-51	2.786e-49	4.79e+02	4.73e+02
GRAP2	6.151e-01	3.380e-51	3.019e-49	4.79e+02	4.79e+02
LAPTM5	6.143e-01	4.895e-51	4.353e-49	4.79e+02	4.79e+02
SUSD3	6.139e-01	5.924e-51	5.245e-49	4.79e+02	4.79e+02
DOCK8	6.136e-01	7.013e-51	6.182e-49	4.79e+02	4.79e+02
FOXP3	6.135e-01	7.200e-51	6.318e-49	4.79e+02	4.79e+02
GPSM3	6.130e-01	9.370e-51	8.188e-49	4.79e+02	4.79e+02
RCSD1	6.128e-01	9.906e-51	8.618e-49	4.79e+02	4.79e+02
INPP5D	6.124e-01	1.232e-50	1.067e-48	4.79e+02	4.79e+02
SLAMF7	6.122e-01	1.331e-50	1.148e-48	4.79e+02	4.79e+02
SPI1	6.119e-01	1.506e-50	1.294e-48	4.79e+02	4.79e+02
EBI3	6.116e-01	1.780e-50	1.523e-48	4.79e+02	4.79e+02
IFIH1	6.116e-01	1.807e-50	1.539e-48	4.79e+02	4.79e+02
SP100	6.111e-01	2.273e-50	1.928e-48	4.79e+02	4.79e+02
PLEK	6.097e-01	4.248e-50	3.587e-48	4.79e+02	4.79e+02
CD300LF	6.097e-01	4.347e-50	3.655e-48	4.79e+02	4.79e+02
CXorf21	6.096e-01	4.556e-50	3.815e-48	4.79e+02	4.79e+02
SLA	6.084e-01	7.681e-50	6.406e-48	4.79e+02	4.79e+02
C16orf54	6.083e-01	8.357e-50	6.940e-48	4.79e+02	4.79e+02
NCF1B	6.078e-01	1.020e-49	8.439e-48	4.79e+02	4.79e+02
SAMD3	6.075e-01	1.188e-49	9.782e-48	4.79e+02	4.75e+02
GVIN1	6.065e-01	1.868e-49	1.532e-47	4.79e+02	4.79e+02
HLA-DOA	6.061e-01	2.302e-49	1.880e-47	4.79e+02	4.79e+02
AIM2	6.052e-01	3.351e-49	2.726e-47	4.79e+02	4.79e+02
KIAA0748	6.042e-01	5.321e-49	4.312e-47	4.79e+02	4.79e+02
CYBB	6.039e-01	6.144e-49	4.959e-47	4.79e+02	4.79e+02
XCL2	6.039e-01	6.230e-49	5.008e-47	4.79e+02	4.79e+02
RTP4	6.032e-01	8.620e-49	6.902e-47	4.79e+02	4.79e+02
FCGR1C	6.025e-01	1.160e-48	9.252e-47	4.79e+02	4.79e+02
CD69	6.025e-01	1.180e-48	9.377e-47	4.79e+02	4.79e+02
FCGR3A	6.024e-01	1.212e-48	9.589e-47	4.79e+02	4.79e+02
MIR155HG	6.024e-01	1.225e-48	9.652e-47	4.79e+02	4.79e+02
CCL3	6.018e-01	1.638e-48	1.286e-46	4.79e+02	4.79e+02
CD4	6.012e-01	2.070e-48	1.619e-46	4.79e+02	4.79e+02
IGSF6	6.006e-01	2.693e-48	2.098e-46	4.79e+02	4.79e+02
TYROBP	5.999e-01	3.782e-48	2.935e-46	4.79e+02	4.79e+02
CD72	5.997e-01	4.209e-48	3.253e-46	4.79e+02	4.79e+02
MYO1F	5.996e-01	4.237e-48	3.262e-46	4.79e+02	4.79e+02
LAIR1	5.995e-01	4.461e-48	3.422e-46	4.79e+02	4.79e+02
ICAM3	5.992e-01	5.243e-48	4.006e-46	4.79e+02	4.79e+02
TMEM150B	5.990e-01	5.738e-48	4.368e-46	4.79e+02	4.75e+02
IL9R	5.985e-01	7.029e-48	5.331e-46	4.79e+02	4.75e+02
P2RY13	5.984e-01	7.472e-48	5.630e-46	4.79e+02	4.79e+02
APOBEC3G	5.984e-01	7.480e-48	5.630e-46	4.79e+02	4.79e+02
EVI2B	5.980e-01	8.721e-48	6.539e-46	4.79e+02	4.79e+02
CYTIP	5.979e-01	9.019e-48	6.738e-46	4.79e+02	4.79e+02
C10orf54	5.975e-01	1.079e-47	8.032e-46	4.79e+02	4.79e+02
TFEC	5.973e-01	1.202e-47	8.913e-46	4.79e+02	4.79e+02
S1PR4	5.962e-01	1.990e-47	1.470e-45	4.79e+02	4.78e+02
DOCK2	5.951e-01	3.131e-47	2.305e-45	4.79e+02	4.79e+02
RGL4	5.947e-01	3.789e-47	2.779e-45	4.79e+02	4.77e+02
APBB1IP	5.945e-01	4.207e-47	3.075e-45	4.79e+02	4.79e+02
STAT5A	5.942e-01	4.690e-47	3.415e-45	4.79e+02	4.79e+02
IL27	5.936e-01	6.114e-47	4.436e-45	4.79e+02	4.07e+02

Target Gene/Attribute	Spearman Correlation	P-value	FDR (BH)	Event_SD	Event_TD
FUT7	5.936e-01	6.137e-47	4.437e-45	4.79e+02	4.77e+02
AKAP5	5.929e-01	8.439e-47	6.079e-45	4.79e+02	4.79e+02
CLEC6A	5.928e-01	8.703e-47	6.247e-45	4.79e+02	4.13e+02
CD200R1	5.926e-01	9.638e-47	6.871e-45	4.79e+02	4.79e+02
SECTM1	5.926e-01	9.641e-47	6.871e-45	4.79e+02	4.79e+02
IKZF1	5.925e-01	9.834e-47	6.984e-45	4.79e+02	4.79e+02
GZMM	5.923e-01	1.108e-46	7.837e-45	4.79e+02	4.78e+02
TNFRSF14	5.921e-01	1.200e-46	8.465e-45	4.79e+02	4.79e+02
NMI	5.914e-01	1.630e-46	1.145e-44	4.79e+02	4.79e+02
SP110	5.910e-01	1.887e-46	1.321e-44	4.79e+02	4.79e+02
CSF2RB	5.910e-01	1.937e-46	1.352e-44	4.79e+02	4.79e+02
IL2RA	5.903e-01	2.555e-46	1.777e-44	4.79e+02	4.79e+02
LOC100233209	5.902e-01	2.735e-46	1.896e-44	4.79e+02	4.79e+02
HLA-DRB6	5.898e-01	3.263e-46	2.253e-44	4.79e+02	4.75e+02
C17orf87	5.896e-01	3.476e-46	2.392e-44	4.79e+02	4.78e+02
IL16	5.895e-01	3.667e-46	2.515e-44	4.79e+02	4.79e+02
RASSF4	5.895e-01	3.732e-46	2.551e-44	4.79e+02	4.79e+02
CD27	5.886e-01	5.399e-46	3.678e-44	4.79e+02	4.79e+02
GPR132	5.884e-01	5.988e-46	4.066e-44	4.79e+02	4.79e+02
MPEG1	5.879e-01	7.218e-46	4.884e-44	4.79e+02	4.79e+02
CCR2	5.876e-01	8.377e-46	5.650e-44	4.79e+02	4.79e+02
GPR114	5.872e-01	1.000e-45	6.724e-44	4.79e+02	4.79e+02
PARP9	5.871e-01	1.030e-45	6.901e-44	4.79e+02	4.79e+02
TLR8	5.871e-01	1.046e-45	6.981e-44	4.79e+02	4.79e+02
GLRX	5.856e-01	1.960e-45	1.304e-43	4.79e+02	4.79e+02
LILRB4	5.852e-01	2.329e-45	1.545e-43	4.79e+02	4.79e+02
SERPINB9	5.841e-01	3.693e-45	2.441e-43	4.79e+02	4.79e+02
NFAM1	5.839e-01	4.106e-45	2.705e-43	4.79e+02	4.79e+02
GJD3	5.832e-01	5.363e-45	3.522e-43	4.79e+02	4.79e+02
MNDA	5.827e-01	6.880e-45	4.504e-43	4.79e+02	4.79e+02
CMKLR1	5.826e-01	7.040e-45	4.594e-43	4.79e+02	4.79e+02
ITGB2	5.823e-01	8.126e-45	5.285e-43	4.79e+02	4.79e+02
TAPBP	5.821e-01	8.857e-45	5.742e-43	4.79e+02	4.79e+02
EVI2A	5.819e-01	9.615e-45	6.213e-43	4.79e+02	4.79e+02
HSH2D	5.818e-01	9.873e-45	6.359e-43	4.79e+02	4.79e+02
AKNA	5.815e-01	1.102e-44	7.074e-43	4.79e+02	4.79e+02
CD40LG	5.813e-01	1.201e-44	7.684e-43	4.79e+02	4.75e+02
MLKL	5.810e-01	1.399e-44	8.929e-43	4.79e+02	4.79e+02
CCRL2	5.809e-01	1.457e-44	9.264e-43	4.79e+02	4.79e+02
TMEM176B	5.808e-01	1.492e-44	9.459e-43	4.79e+02	4.79e+02
TLR3	5.808e-01	1.529e-44	9.661e-43	4.79e+02	4.79e+02
FMNL1	5.795e-01	2.537e-44	1.599e-42	4.79e+02	4.79e+02
LRRC25	5.794e-01	2.714e-44	1.705e-42	4.79e+02	4.79e+02
CD37	5.793e-01	2.754e-44	1.724e-42	4.79e+02	4.79e+02
TMEM149	5.790e-01	3.130e-44	1.954e-42	4.79e+02	4.79e+02
TTC24	5.782e-01	4.490e-44	2.794e-42	4.79e+02	4.22e+02
MS4A6A	5.778e-01	5.354e-44	3.321e-42	4.79e+02	4.79e+02
CD52	5.773e-01	6.476e-44	4.004e-42	4.79e+02	4.79e+02
KMO	5.770e-01	7.374e-44	4.546e-42	4.79e+02	4.79e+02
C17orf60	5.765e-01	8.961e-44	5.507e-42	4.79e+02	4.77e+02
BTK	5.758e-01	1.214e-43	7.439e-42	4.79e+02	4.79e+02
LYZ	5.734e-01	3.237e-43	1.977e-41	4.79e+02	4.79e+02
C3AR1	5.728e-01	4.039e-43	2.459e-41	4.79e+02	4.79e+02
BCL2L14	5.722e-01	5.312e-43	3.225e-41	4.79e+02	4.78e+02
HLA-DQA1	5.720e-01	5.700e-43	3.450e-41	4.79e+02	4.79e+02
CLEC4A	5.717e-01	6.534e-43	3.943e-41	4.79e+02	4.79e+02

Target Gene/Attribute	Spearman Correlation	P-value	FDR (BH)	Event_SD	Event_TD
DOK3	5.716e-01	6.830e-43	4.110e-41	4.79e+02	4.79e+02
HAPLN3	5.715e-01	6.907e-43	4.143e-41	4.79e+02	4.79e+02
ARHGAP30	5.715e-01	6.977e-43	4.173e-41	4.79e+02	4.79e+02
CD97	5.715e-01	7.020e-43	4.186e-41	4.79e+02	4.79e+02
FAM78A	5.715e-01	7.077e-43	4.208e-41	4.79e+02	4.79e+02
RNASE6	5.710e-01	8.531e-43	5.058e-41	4.79e+02	4.79e+02
SRGN	5.708e-01	9.190e-43	5.432e-41	4.79e+02	4.79e+02
C1orf162	5.707e-01	9.536e-43	5.620e-41	4.79e+02	4.79e+02
ZNF831	5.703e-01	1.129e-42	6.636e-41	4.79e+02	4.78e+02
CCR1	5.700e-01	1.304e-42	7.638e-41	4.79e+02	4.79e+02
CDC42SE2	5.696e-01	1.540e-42	8.996e-41	4.79e+02	4.79e+02
LGALS9	5.695e-01	1.605e-42	9.350e-41	4.79e+02	4.79e+02
ACY3	5.694e-01	1.646e-42	9.560e-41	4.79e+02	4.77e+02
C2	5.691e-01	1.858e-42	1.076e-40	4.79e+02	4.79e+02
NCR1	5.683e-01	2.515e-42	1.453e-40	4.79e+02	3.80e+02
FPR3	5.683e-01	2.599e-42	1.497e-40	4.79e+02	4.79e+02
ADAMDEC1	5.669e-01	4.562e-42	2.620e-40	4.79e+02	4.79e+02
PML	5.658e-01	7.035e-42	4.028e-40	4.79e+02	4.79e+02
SERPING1	5.657e-01	7.248e-42	4.138e-40	4.79e+02	4.79e+02
GPR84	5.655e-01	7.906e-42	4.501e-40	4.79e+02	4.79e+02
TRIM34	5.649e-01	1.001e-41	5.684e-40	4.79e+02	4.79e+02
BST2	5.648e-01	1.018e-41	5.762e-40	4.79e+02	4.79e+02
C5orf20	5.647e-01	1.091e-41	6.156e-40	4.79e+02	4.76e+02
PARP12	5.643e-01	1.242e-41	6.990e-40	4.79e+02	4.79e+02
CD300A	5.643e-01	1.257e-41	7.054e-40	4.79e+02	4.79e+02
JAK2	5.642e-01	1.319e-41	7.385e-40	4.79e+02	4.79e+02
SIGLEC7	5.640e-01	1.411e-41	7.880e-40	4.79e+02	4.79e+02
BCL2A1	5.637e-01	1.603e-41	8.922e-40	4.79e+02	4.79e+02
CSF2RA	5.635e-01	1.761e-41	9.777e-40	4.79e+02	4.79e+02
PLEKHO2	5.628e-01	2.321e-41	1.285e-39	4.79e+02	4.79e+02
PLA2G7	5.626e-01	2.460e-41	1.358e-39	4.79e+02	4.79e+02
JAK3	5.625e-01	2.571e-41	1.416e-39	4.79e+02	4.79e+02
GPBAR1	5.620e-01	3.078e-41	1.690e-39	4.79e+02	4.79e+02
LOC400696	5.611e-01	4.392e-41	2.405e-39	4.79e+02	4.23e+02
CMPK2	5.611e-01	4.454e-41	2.432e-39	4.79e+02	4.79e+02
CLEC4E	5.609e-01	4.739e-41	2.581e-39	4.79e+02	4.79e+02
C1orf38	5.599e-01	7.009e-41	3.807e-39	4.79e+02	4.79e+02
SPOCK2	5.598e-01	7.446e-41	4.034e-39	4.79e+02	4.79e+02
TYMP	5.585e-01	1.212e-40	6.545e-39	4.79e+02	4.79e+02
STAC3	5.577e-01	1.662e-40	8.955e-39	4.79e+02	4.79e+02
RAC2	5.574e-01	1.892e-40	1.016e-38	4.79e+02	4.79e+02
CASP5	5.562e-01	2.960e-40	1.586e-38	4.79e+02	4.54e+02
PPP1R16B	5.562e-01	2.970e-40	1.588e-38	4.79e+02	4.79e+02
IFI44L	5.556e-01	3.807e-40	2.030e-38	4.79e+02	4.79e+02
NCR3	5.554e-01	4.145e-40	2.204e-38	4.79e+02	4.73e+02
DTX3L	5.550e-01	4.803e-40	2.547e-38	4.79e+02	4.79e+02
NFKBIE	5.548e-01	5.219e-40	2.760e-38	4.79e+02	4.79e+02
FAS	5.538e-01	7.638e-40	4.029e-38	4.79e+02	4.79e+02
ISG20	5.532e-01	9.440e-40	4.967e-38	4.79e+02	4.79e+02
PARVG	5.531e-01	9.699e-40	5.089e-38	4.79e+02	4.79e+02
CCR8	5.527e-01	1.166e-39	6.102e-38	4.79e+02	4.75e+02
CCR7	5.524e-01	1.267e-39	6.613e-38	4.79e+02	4.79e+02
PIK3CG	5.523e-01	1.319e-39	6.866e-38	4.79e+02	4.79e+02
CD8B	5.519e-01	1.556e-39	8.081e-38	4.79e+02	4.79e+02
WIPF1	5.519e-01	1.574e-39	8.153e-38	4.79e+02	4.79e+02
GPR25	5.518e-01	1.627e-39	8.403e-38	4.79e+02	3.90e+02

Target Gene/Attribute	Spearman Correlation	P-value	FDR (BH)	Event_SD	Event_TD
MMP25	5.515e-01	1.832e-39	9.443e-38	4.79e+02	4.79e+02
RAB33A	5.510e-01	2.170e-39	1.115e-37	4.79e+02	4.79e+02
CSF1R	5.509e-01	2.296e-39	1.177e-37	4.79e+02	4.79e+02
TRPV2	5.508e-01	2.397e-39	1.226e-37	4.79e+02	4.79e+02
MFNG	5.506e-01	2.504e-39	1.277e-37	4.79e+02	4.79e+02
TMEM140	5.502e-01	2.913e-39	1.482e-37	4.79e+02	4.79e+02
CEACAM21	5.501e-01	3.038e-39	1.542e-37	4.79e+02	4.78e+02
ARHGEF6	5.500e-01	3.208e-39	1.624e-37	4.79e+02	4.79e+02
BTLA	5.499e-01	3.272e-39	1.652e-37	4.79e+02	4.78e+02
PDCD1LG2	5.495e-01	3.834e-39	1.931e-37	4.79e+02	4.79e+02
TICAM2	5.486e-01	5.483e-39	2.755e-37	4.79e+02	4.73e+02
TLR7	5.479e-01	7.080e-39	3.548e-37	4.79e+02	4.79e+02
NR1H3	5.477e-01	7.630e-39	3.814e-37	4.79e+02	4.79e+02
CD28	5.474e-01	8.534e-39	4.256e-37	4.79e+02	4.79e+02
CD14	5.468e-01	1.061e-38	5.277e-37	4.79e+02	4.79e+02
LAT	5.464e-01	1.223e-38	6.070e-37	4.79e+02	4.79e+02
KLRC4	5.463e-01	1.265e-38	6.263e-37	4.79e+02	3.66e+02
HCG26	5.462e-01	1.340e-38	6.619e-37	4.79e+02	4.79e+02
HLA-DQA2	5.461e-01	1.404e-38	6.918e-37	4.79e+02	4.79e+02
PATL2	5.459e-01	1.472e-38	7.233e-37	4.79e+02	4.73e+02
COTL1	5.455e-01	1.710e-38	8.383e-37	4.79e+02	4.79e+02
CD84	5.453e-01	1.862e-38	9.104e-37	4.79e+02	4.79e+02
ICAM1	5.449e-01	2.136e-38	1.042e-36	4.79e+02	4.79e+02
AGAP2	5.444e-01	2.577e-38	1.254e-36	4.79e+02	4.79e+02
ARL6IP5	5.443e-01	2.701e-38	1.311e-36	4.79e+02	4.79e+02
DDX60	5.438e-01	3.286e-38	1.591e-36	4.79e+02	4.79e+02
LTB	5.436e-01	3.453e-38	1.668e-36	4.79e+02	4.79e+02
FAM113B	5.431e-01	4.162e-38	2.006e-36	4.79e+02	4.79e+02
TMEM176A	5.431e-01	4.194e-38	2.017e-36	4.79e+02	4.79e+02
MAT2B	5.429e-01	4.470e-38	2.144e-36	4.79e+02	4.79e+02
PRKCB	5.427e-01	4.895e-38	2.342e-36	4.79e+02	4.79e+02
CD300C	5.426e-01	5.065e-38	2.418e-36	4.79e+02	4.79e+02
MYO1G	5.425e-01	5.211e-38	2.481e-36	4.79e+02	4.79e+02
HK3	5.424e-01	5.530e-38	2.627e-36	4.79e+02	4.79e+02
P2RY8	5.422e-01	5.799e-38	2.749e-36	4.79e+02	4.79e+02
STAT4	5.409e-01	9.478e-38	4.482e-36	4.79e+02	4.79e+02
TBXAS1	5.405e-01	1.115e-37	5.262e-36	4.79e+02	4.79e+02
TNFSF12-TNFSF13	5.402e-01	1.241e-37	5.840e-36	4.79e+02	4.63e+02
PILRA	5.396e-01	1.505e-37	7.067e-36	4.79e+02	4.79e+02
OAS2	5.393e-01	1.673e-37	7.836e-36	4.79e+02	4.79e+02
AMICA1	5.393e-01	1.696e-37	7.925e-36	4.79e+02	4.79e+02
ARHGAP25	5.379e-01	2.836e-37	1.322e-35	4.79e+02	4.79e+02
SIGLEC9	5.378e-01	2.918e-37	1.358e-35	4.79e+02	4.79e+02
KCNJ10	5.371e-01	3.734e-37	1.733e-35	4.79e+02	4.77e+02
DOCK10	5.356e-01	6.583e-37	3.049e-35	4.79e+02	4.79e+02
FLI1	5.354e-01	7.062e-37	3.263e-35	4.79e+02	4.79e+02
LY86	5.352e-01	7.412e-37	3.416e-35	4.79e+02	4.79e+02
EMR1	5.349e-01	8.284e-37	3.810e-35	4.79e+02	4.77e+02
LILRB3	5.336e-01	1.336e-36	6.130e-35	4.79e+02	4.79e+02
PHF11	5.336e-01	1.348e-36	6.172e-35	4.79e+02	4.79e+02
ADAM8	5.334e-01	1.422e-36	6.497e-35	4.79e+02	4.79e+02
SH2D2A	5.314e-01	2.932e-36	1.336e-34	4.79e+02	4.79e+02
GIMAP8	5.313e-01	3.028e-36	1.377e-34	4.79e+02	4.79e+02
NECAP2	5.306e-01	3.930e-36	1.783e-34	4.79e+02	4.79e+02
GAB3	5.299e-01	4.913e-36	2.224e-34	4.79e+02	4.79e+02
ZMYND15	5.297e-01	5.273e-36	2.382e-34	4.79e+02	4.79e+02

Target Gene/Attribute	Spearman Correlation	P-value	FDR (BH)	Event_SD	Event_TD
LILRA6	5.297e-01	5.366e-36	2.418e-34	4.79e+02	4.78e+02
RGS18	5.293e-01	6.096e-36	2.741e-34	4.79e+02	4.79e+02
N4BP2L1	5.292e-01	6.319e-36	2.835e-34	4.79e+02	4.79e+02
LOC100129066	5.288e-01	7.227e-36	3.235e-34	4.79e+02	4.20e+02
LILRP2	5.282e-01	9.121e-36	4.074e-34	4.79e+02	3.86e+02
PLA2G2D	5.277e-01	1.094e-35	4.875e-34	4.79e+02	4.79e+02
LOC606724	5.275e-01	1.143e-35	5.081e-34	4.79e+02	4.79e+02
CCR4	5.273e-01	1.227e-35	5.446e-34	4.79e+02	4.72e+02
CSF1	5.273e-01	1.248e-35	5.524e-34	4.79e+02	4.79e+02
ALOX5	5.270e-01	1.381e-35	6.099e-34	4.79e+02	4.79e+02
CARD9	5.268e-01	1.473e-35	6.494e-34	4.79e+02	4.79e+02
LSP1	5.266e-01	1.577e-35	6.933e-34	4.79e+02	4.79e+02
SAMHD1	5.265e-01	1.619e-35	7.103e-34	4.79e+02	4.79e+02
TNFSF8	5.261e-01	1.879e-35	8.226e-34	4.79e+02	4.78e+02
CR1L	5.253e-01	2.502e-35	1.093e-33	4.79e+02	3.99e+02
MEFV	5.253e-01	2.510e-35	1.094e-33	4.79e+02	4.79e+02
TCIRG1	5.251e-01	2.672e-35	1.162e-33	4.79e+02	4.79e+02
DPF3	5.249e-01	2.827e-35	1.227e-33	4.79e+02	4.78e+02
IFIT2	5.242e-01	3.667e-35	1.588e-33	4.79e+02	4.79e+02
GSDMD	5.238e-01	4.152e-35	1.794e-33	4.79e+02	4.79e+02
SIRPB2	5.232e-01	5.241e-35	2.260e-33	4.79e+02	4.79e+02
GPR141	5.229e-01	5.684e-35	2.446e-33	4.79e+02	4.65e+02
CLECL1	5.226e-01	6.292e-35	2.702e-33	4.79e+02	4.71e+02
LCP1	5.219e-01	7.989e-35	3.423e-33	4.79e+02	4.79e+02
LY9	5.215e-01	9.176e-35	3.924e-33	4.79e+02	4.79e+02
GPR183	5.214e-01	9.445e-35	4.030e-33	4.79e+02	4.79e+02
MAP4K1	5.208e-01	1.190e-34	5.065e-33	4.79e+02	4.79e+02
C15orf53	5.207e-01	1.238e-34	5.258e-33	4.79e+02	3.07e+02
GGTA1	5.206e-01	1.240e-34	5.258e-33	4.79e+02	4.79e+02
TNFSF14	5.206e-01	1.263e-34	5.343e-33	4.79e+02	4.72e+02
SLCO2B1	5.202e-01	1.437e-34	6.067e-33	4.79e+02	4.79e+02
PIK3R5	5.194e-01	1.897e-34	7.991e-33	4.79e+02	4.79e+02
PIK3R6	5.193e-01	1.947e-34	8.188e-33	4.79e+02	4.79e+02
CD33	5.192e-01	2.053e-34	8.613e-33	4.79e+02	4.79e+02
CFB	5.191e-01	2.099e-34	8.787e-33	4.79e+02	4.79e+02
FGR	5.190e-01	2.153e-34	8.995e-33	4.79e+02	4.79e+02
CLEC4D	5.190e-01	2.167e-34	9.033e-33	4.79e+02	4.58e+02
MEI1	5.183e-01	2.772e-34	1.153e-32	4.79e+02	4.79e+02
IRF4	5.182e-01	2.901e-34	1.205e-32	4.79e+02	4.79e+02
PVRIG	5.180e-01	3.069e-34	1.272e-32	4.79e+02	4.79e+02
FCRL3	5.167e-01	4.814e-34	1.991e-32	4.79e+02	4.75e+02
ALOX5AP	5.163e-01	5.511e-34	2.274e-32	4.79e+02	4.79e+02
TMEM106A	5.154e-01	7.325e-34	3.017e-32	4.79e+02	4.79e+02
FAIM3	5.152e-01	7.987e-34	3.283e-32	4.79e+02	4.79e+02
CXCL13	5.145e-01	9.932e-34	4.074e-32	4.79e+02	4.79e+02
GRIN3A	5.145e-01	9.978e-34	4.084e-32	4.79e+02	4.78e+02
TRIM69	5.141e-01	1.123e-33	4.589e-32	4.79e+02	4.79e+02
SIGLEC1	5.134e-01	1.454e-33	5.929e-32	4.79e+02	4.79e+02
SIGLEC8	5.133e-01	1.478e-33	6.014e-32	4.79e+02	4.77e+02
CYBA	5.129e-01	1.716e-33	6.968e-32	4.79e+02	4.79e+02
C14orf73	5.128e-01	1.739e-33	7.047e-32	4.79e+02	4.77e+02
HLA-DOB	5.128e-01	1.748e-33	7.067e-32	4.79e+02	4.79e+02
STK10	5.128e-01	1.776e-33	7.168e-32	4.79e+02	4.79e+02
TNFRSF4	5.124e-01	2.046e-33	8.240e-32	4.79e+02	4.78e+02
TNIP1	5.119e-01	2.388e-33	9.598e-32	4.79e+02	4.79e+02
