# Supplementary material for: A Multi-Level miRNA Regulatory Network Associated with IRF1 Expression in Non-Small Cell Lung Cancer: In Silico Identification of Candidate Biomarkers for Immunotherapy Response
Source: Int J Mol Sci. 2026 Jun 8;27(12):5192. doi: 10.3390/ijms27125192 (PMC13300628; doi:10.3390/ijms27125192)
Supplement: Supplementary file 1 [file ijms-27-05192-s001.zip › ijms-4286133-supplementary/Supplementary Table S3.pdf]

Supplementary Table S3. List of microRNAs with the highest negative correlation coefficient with the IRF1 gene level in LUAD.

Target Gene/Attribute	Spearman Correlation	P-value	FDR (BH)	Event_SD	Event_TD
hsa-mir-200b	-3.708e-01	3.969e-10	3.515e-08	2.71e+02	2.71e+02
hsa-mir-429	-3.058e-01	3.250e-07	1.363e-05	2.71e+02	2.71e+02
hsa-mir-200a	-2.905e-01	1.273e-06	4.612e-05	2.71e+02	2.71e+02
hsa-mir-3189	-2.807e-01	2.668e-06	8.505e-05	2.71e+02	2.01e+02
hsa-mir-375	-2.768e-01	4.069e-06	1.158e-04	2.71e+02	2.71e+02
hsa-mir-1287	-2.728e-01	5.650e-06	1.549e-04	2.71e+02	2.71e+02
hsa-mir-182	-2.647e-01	1.081e-05	2.693e-04	2.71e+02	2.71e+02
hsa-mir-183	-2.598e-01	1.581e-05	3.706e-04	2.71e+02	2.71e+02
hsa-mir-135b	-2.490e-01	3.597e-05	7.350e-04	2.71e+02	2.71e+02
hsa-mir-200c	-2.425e-01	5.774e-05	1.151e-03	2.71e+02	2.71e+02
hsa-mir-3177	-2.403e-01	6.454e-05	1.221e-03	2.71e+02	1.52e+02
hsa-mir-877	-2.300e-01	1.331e-04	2.210e-03	2.71e+02	2.69e+02
hsa-mir-1266	-2.228e-01	2.259e-04	3.531e-03	2.71e+02	2.71e+02
hsa-mir-3200	-2.135e-01	4.022e-04	5.724e-03	2.71e+02	2.67e+02
hsa-mir-548v	-2.036e-01	7.480e-04	9.463e-03	2.71e+02	2.55e+02
hsa-mir-99b	-2.026e-01	8.142e-04	1.009e-02	2.71e+02	2.71e+02
hsa-mir-552	-2.018e-01	8.334e-04	1.009e-02	2.71e+02	2.13e+02
hsa-mir-96	-2.014e-01	8.760e-04	1.042e-02	2.71e+02	2.71e+02
hsa-mir-3065	-2.001e-01	9.428e-04	1.089e-02	2.71e+02	2.71e+02
hsa-mir-1275	-1.860e-01	2.109e-03	2.271e-02	2.71e+02	1.88e+02
hsa-mir-1180	-1.857e-01	2.171e-03	2.307e-02	2.71e+02	2.71e+02
hsa-mir-3660	-1.833e-01	2.457e-03	2.543e-02	2.71e+02	6.50e+01
hsa-mir-181d	-1.808e-01	2.859e-03	2.848e-02	2.71e+02	2.71e+02
hsa-mir-301b	-1.787e-01	3.165e-03	3.114e-02	2.71e+02	2.57e+02
hsa-mir-3127	-1.742e-01	4.066e-03	3.768e-02	2.71e+02	2.71e+02
hsa-mir-548b	-1.733e-01	4.214e-03	3.860e-02	2.71e+02	2.58e+02
hsa-mir-1276	-1.719e-01	4.539e-03	4.111e-02	2.71e+02	1.48e+02
hsa-mir-559	-1.705e-01	4.875e-03	4.317e-02	2.71e+02	1.35e+02
hsa-mir-30d	-1.698e-01	5.120e-03	4.484e-02	2.71e+02	2.71e+02
hsa-mir-556	-1.680e-01	5.550e-03	4.756e-02	2.71e+02	2.36e+02
hsa-mir-219-1	-1.671e-01	5.872e-03	4.949e-02	2.71e+02	2.70e+02
hsa-mir-578	-1.669e-01	5.899e-03	4.949e-02	2.71e+02	9.80e+01
hsa-mir-148b	-1.654e-01	6.388e-03	5.303e-02	2.71e+02	2.71e+02
hsa-mir-141	-1.636e-01	7.003e-03	5.754e-02	2.71e+02	2.71e+02
hsa-mir-187	-1.627e-01	7.272e-03	5.848e-02	2.71e+02	2.67e+02
hsa-mir-653	-1.590e-01	8.782e-03	6.730e-02	2.71e+02	2.71e+02
hsa-mir-3130-2	-1.572e-01	9.537e-03	6.974e-02	2.71e+02	1.10e+01
hsa-mir-613	-1.569e-01	9.664e-03	6.974e-02	2.71e+02	4.00e+00
hsa-mir-615	-1.569e-01	9.682e-03	6.974e-02	2.71e+02	2.12e+02
hsa-mir-216a	-1.568e-01	9.751e-03	6.974e-02	2.71e+02	2.24e+02
hsa-mir-3170	-1.541e-01	1.107e-02	7.672e-02	2.71e+02	2.50e+02
hsa-let-7e	-1.540e-01	1.119e-02	7.686e-02	2.71e+02	2.71e+02
