# Supplementary material for: A Multi-Level miRNA Regulatory Network Associated with IRF1 Expression in Non-Small Cell Lung Cancer: In Silico Identification of Candidate Biomarkers for Immunotherapy Response
Source: Int J Mol Sci. 2026 Jun 8;27(12):5192. doi: 10.3390/ijms27125192 (PMC13300628; doi:10.3390/ijms27125192)
Supplement: Supplementary file 1 [file ijms-27-05192-s001.zip › ijms-4286133-supplementary/Supplementary Table S4.pdf]

Supplementary Table S4. List of microRNAs with the highest negative correlation coefficient with the IRF1 gene level in LUSC.

Target Gene/Attribute	Spearman Correlation	P-value	FDR (BH)	Event_SD	Event_TD
hsa-mir-3200	-4.336e-01	2.229e-16	3.642e-14	3.26e+02	3.24e+02
hsa-mir-183	-4.118e-01	8.966e-15	1.221e-12	3.26e+02	3.26e+02
hsa-mir-944	-4.048e-01	2.783e-14	3.248e-12	3.26e+02	3.24e+02
hsa-mir-301b	-3.997e-01	6.198e-14	6.330e-12	3.26e+02	3.24e+02
hsa-mir-1296	-3.951e-01	1.260e-13	1.144e-11	3.26e+02	3.26e+02
hsa-mir-1180	-3.821e-01	8.998e-13	7.352e-11	3.26e+02	3.26e+02
hsa-mir-130b	-3.784e-01	1.536e-12	1.141e-10	3.26e+02	3.26e+02
hsa-mir-219-1	-3.667e-01	8.154e-12	5.551e-10	3.26e+02	3.25e+02
hsa-mir-33a	-3.552e-01	3.941e-11	2.477e-09	3.26e+02	3.26e+02
hsa-mir-96	-3.545e-01	4.384e-11	2.558e-09	3.26e+02	3.26e+02
hsa-mir-561	-3.517e-01	6.312e-11	3.438e-09	3.26e+02	2.71e+02
hsa-mir-205	-3.488e-01	9.234e-11	4.715e-09	3.26e+02	3.26e+02
hsa-mir-324	-3.463e-01	1.289e-10	6.193e-09	3.26e+02	3.26e+02
hsa-mir-767	-3.457e-01	1.387e-10	6.298e-09	3.26e+02	3.10e+02
hsa-mir-429	-3.350e-01	5.427e-10	2.334e-08	3.26e+02	3.26e+02
hsa-mir-182	-3.323e-01	7.597e-10	3.103e-08	3.26e+02	3.26e+02
hsa-mir-128-1	-3.287e-01	1.194e-09	4.645e-08	3.26e+02	3.26e+02
hsa-mir-105-1	-3.221e-01	2.636e-09	9.788e-08	3.26e+02	2.94e+02
hsa-mir-200b	-3.178e-01	4.379e-09	1.555e-07	3.26e+02	3.26e+02
hsa-mir-744	-3.172e-01	4.693e-09	1.598e-07	3.26e+02	3.26e+02
hsa-mir-197	-3.160e-01	5.388e-09	1.761e-07	3.26e+02	3.26e+02
hsa-mir-1306	-3.149e-01	6.143e-09	1.930e-07	3.26e+02	3.26e+02
hsa-mir-128-2	-3.145e-01	6.468e-09	1.957e-07	3.26e+02	3.26e+02
hsa-mir-105-2	-3.131e-01	7.588e-09	2.214e-07	3.26e+02	2.99e+02
hsa-mir-466	-3.118e-01	8.779e-09	2.473e-07	3.26e+02	1.78e+02
hsa-mir-93	-3.062e-01	1.671e-08	4.552e-07	3.26e+02	3.26e+02
hsa-mir-877	-3.007e-01	3.075e-08	8.105e-07	3.26e+02	3.21e+02
hsa-mir-421	-2.997e-01	3.416e-08	8.721e-07	3.26e+02	3.23e+02
hsa-mir-103-1	-2.954e-01	5.505e-08	1.363e-06	3.26e+02	3.26e+02
hsa-mir-671	-2.928e-01	7.274e-08	1.748e-06	3.26e+02	3.26e+02
hsa-mir-423	-2.918e-01	8.109e-08	1.893e-06	3.26e+02	3.26e+02
hsa-mir-708	-2.829e-01	2.047e-07	4.646e-06	3.26e+02	3.26e+02
hsa-mir-187	-2.803e-01	2.678e-07	5.912e-06	3.26e+02	3.23e+02
hsa-mir-200a	-2.771e-01	3.699e-07	7.954e-06	3.26e+02	3.26e+02
hsa-mir-345	-2.748e-01	4.636e-07	9.711e-06	3.26e+02	3.26e+02
hsa-mir-98	-2.728e-01	5.673e-07	1.159e-05	3.26e+02	3.26e+02
hsa-mir-548v	-2.681e-01	9.021e-07	1.798e-05	3.26e+02	3.14e+02
hsa-mir-28	-2.651e-01	1.202e-06	2.339e-05	3.26e+02	3.26e+02
hsa-mir-1910	-2.628e-01	1.496e-06	2.778e-05	3.26e+02	2.77e+02
hsa-mir-3170	-2.613e-01	1.720e-06	3.006e-05	3.26e+02	3.06e+02
hsa-mir-532	-2.613e-01	1.729e-06	3.006e-05	3.26e+02	3.26e+02
hsa-mir-301a	-2.609e-01	1.797e-06	3.058e-05	3.26e+02	3.26e+02
hsa-mir-1226	-2.603e-01	1.903e-06	3.173e-05	3.26e+02	3.19e+02
hsa-mir-500a	-2.575e-01	2.464e-06	3.871e-05	3.26e+02	3.26e+02
hsa-mir-1262	-2.558e-01	2.891e-06	4.456e-05	3.26e+02	3.05e+02
hsa-mir-378	-2.531e-01	3.708e-06	5.610e-05	3.26e+02	3.26e+02
hsa-mir-9-1	-2.516e-01	4.233e-06	6.287e-05	3.26e+02	3.26e+02
hsa-mir-9-2	-2.514e-01	4.311e-06	6.289e-05	3.26e+02	3.26e+02
hsa-mir-2277	-2.501e-01	4.838e-06	6.935e-05	3.26e+02	3.21e+02
hsa-mir-452	-2.495e-01	5.125e-06	7.220e-05	3.26e+02	3.26e+02
hsa-mir-185	-2.483e-01	5.682e-06	7.868e-05	3.26e+02	3.26e+02
hsa-mir-335	-2.480e-01	5.831e-06	7.940e-05	3.26e+02	3.26e+02
hsa-mir-200c	-2.449e-01	7.689e-06	9.971e-05	3.26e+02	3.26e+02

Target Gene/Attribute	Spearman Correlation	P-value	FDR (BH)	Event_SD	Event_TD
hsa-mir-3127	-2.448e-01	7.812e-06	9.972e-05	3.26e+02	3.26e+02
hsa-mir-25	-2.436e-01	8.653e-06	1.071e-04	3.26e+02	3.26e+02
hsa-mir-18a	-2.432e-01	8.971e-06	1.084e-04	3.26e+02	3.26e+02
hsa-mir-1292	-2.431e-01	9.063e-06	1.084e-04	3.26e+02	2.97e+02
hsa-mir-106b	-2.402e-01	1.158e-05	1.351e-04	3.26e+02	3.26e+02
hsa-mir-769	-2.393e-01	1.254e-05	1.443e-04	3.26e+02	3.26e+02
hsa-mir-141	-2.379e-01	1.415e-05	1.584e-04	3.26e+02	3.26e+02
hsa-mir-1248	-2.355e-01	1.742e-05	1.923e-04	3.26e+02	3.22e+02
hsa-mir-570	-2.343e-01	1.922e-05	2.015e-04	3.26e+02	2.89e+02
hsa-mir-149	-2.343e-01	1.923e-05	2.015e-04	3.26e+02	3.26e+02
hsa-mir-203	-2.340e-01	1.966e-05	2.033e-04	3.26e+02	3.26e+02
hsa-mir-129-2	-2.337e-01	2.024e-05	2.067e-04	3.26e+02	3.18e+02
hsa-mir-1224	-2.304e-01	2.666e-05	2.689e-04	3.26e+02	2.58e+02
hsa-mir-129-1	-2.294e-01	2.889e-05	2.878e-04	3.26e+02	3.19e+02
hsa-mir-3684	-2.283e-01	3.175e-05	3.088e-04	3.26e+02	2.61e+02
hsa-mir-577	-2.281e-01	3.215e-05	3.090e-04	3.26e+02	3.16e+02
hsa-mir-27b	-2.279e-01	3.262e-05	3.099e-04	3.26e+02	3.26e+02
hsa-mir-500b	-2.274e-01	3.398e-05	3.191e-04	3.26e+02	3.26e+02
hsa-mir-103-2	-2.265e-01	3.658e-05	3.361e-04	3.26e+02	3.26e+02
hsa-mir-1276	-2.265e-01	3.661e-05	3.361e-04	3.26e+02	1.97e+02
hsa-mir-579	-2.263e-01	3.733e-05	3.388e-04	3.26e+02	2.98e+02
hsa-mir-92a-2	-2.256e-01	3.937e-05	3.534e-04	3.26e+02	3.26e+02
hsa-mir-17	-2.245e-01	4.324e-05	3.840e-04	3.26e+02	3.26e+02
hsa-mir-505	-2.218e-01	5.359e-05	4.658e-04	3.26e+02	3.26e+02
hsa-mir-590	-2.214e-01	5.529e-05	4.755e-04	3.26e+02	3.26e+02
hsa-mir-210	-2.203e-01	6.038e-05	5.139e-04	3.26e+02	3.26e+02
hsa-mir-627	-2.175e-01	7.501e-05	6.185e-04	3.26e+02	3.20e+02
hsa-mir-675	-2.173e-01	7.606e-05	6.185e-04	3.26e+02	3.24e+02
hsa-mir-9-3	-2.133e-01	1.039e-04	8.322e-04	3.26e+02	2.81e+02
hsa-mir-660	-2.125e-01	1.103e-04	8.750e-04	3.26e+02	3.26e+02
hsa-mir-574	-2.117e-01	1.174e-04	9.223e-04	3.26e+02	3.26e+02
hsa-mir-196b	-2.114e-01	1.202e-04	9.353e-04	3.26e+02	3.26e+02
hsa-mir-651	-2.113e-01	1.215e-04	9.367e-04	3.26e+02	3.25e+02
hsa-mir-20a	-2.099e-01	1.347e-04	1.024e-03	3.26e+02	3.26e+02
hsa-mir-641	-2.097e-01	1.362e-04	1.024e-03	3.26e+02	1.79e+02
hsa-mir-224	-2.097e-01	1.366e-04	1.024e-03	3.26e+02	3.26e+02
hsa-mir-190	-2.094e-01	1.400e-04	1.040e-03	3.26e+02	3.24e+02
hsa-mir-3691	-2.069e-01	1.682e-04	1.227e-03	3.26e+02	2.63e+02
hsa-mir-556	-2.061e-01	1.788e-04	1.293e-03	3.26e+02	2.49e+02
hsa-mir-3651	-2.052e-01	1.916e-04	1.373e-03	3.26e+02	3.14e+02
hsa-mir-188	-2.027e-01	2.302e-04	1.622e-03	3.26e+02	3.26e+02
hsa-mir-24-2	-2.027e-01	2.303e-04	1.622e-03	3.26e+02	3.26e+02
hsa-mir-1271	-2.020e-01	2.422e-04	1.691e-03	3.26e+02	3.26e+02
hsa-mir-362	-2.015e-01	2.500e-04	1.731e-03	3.26e+02	3.26e+02
hsa-mir-501	-2.000e-01	2.781e-04	1.909e-03	3.26e+02	3.26e+02
hsa-mir-151	-1.944e-01	4.138e-04	2.771e-03	3.26e+02	3.26e+02
hsa-mir-502	-1.936e-01	4.393e-04	2.918e-03	3.26e+02	3.26e+02
hsa-mir-3144	-1.932e-01	4.526e-04	2.982e-03	3.26e+02	1.12e+02
hsa-mir-3065	-1.921e-01	4.864e-04	3.173e-03	3.26e+02	3.26e+02
hsa-mir-934	-1.920e-01	4.893e-04	3.173e-03	3.26e+02	2.85e+02

Target Gene/Attribute	Spearman Correlation	P-value	FDR (BH)	Event_SD	Event_TD
hsa-mir-760	-1.909e-01	5.289e-04	3.403e-03	3.26e+02	3.21e+02
hsa-mir-30b	-1.906e-01	5.397e-04	3.445e-03	3.26e+02	3.26e+02
hsa-mir-629	-1.873e-01	6.777e-04	4.227e-03	3.26e+02	3.26e+02
hsa-mir-15b	-1.838e-01	8.556e-04	5.216e-03	3.26e+02	3.26e+02
hsa-mir-204	-1.828e-01	9.118e-04	5.410e-03	3.26e+02	3.14e+02
hsa-mir-107	-1.828e-01	9.138e-04	5.410e-03	3.26e+02	3.26e+02
hsa-mir-1291	-1.815e-01	9.960e-04	5.854e-03	3.26e+02	2.88e+02
hsa-mir-3199-2	-1.808e-01	1.042e-03	6.078e-03	3.26e+02	2.90e+02
hsa-mir-1914	-1.781e-01	1.239e-03	7.180e-03	3.26e+02	1.32e+02
hsa-mir-624	-1.776e-01	1.279e-03	7.306e-03	3.26e+02	3.20e+02
hsa-mir-3187	-1.773e-01	1.305e-03	7.403e-03	3.26e+02	2.37e+02
hsa-mir-339	-1.762e-01	1.405e-03	7.864e-03	3.26e+02	3.26e+02
hsa-mir-1246	-1.735e-01	1.663e-03	9.058e-03	3.26e+02	1.39e+02
hsa-mir-1301	-1.729e-01	1.728e-03	9.352e-03	3.26e+02	3.26e+02
hsa-mir-412	-1.725e-01	1.771e-03	9.519e-03	3.26e+02	3.14e+02
hsa-mir-3171	-1.696e-01	2.122e-03	1.133e-02	3.26e+02	1.08e+02
hsa-mir-152	-1.685e-01	2.266e-03	1.202e-02	3.26e+02	3.26e+02
hsa-mir-589	-1.677e-01	2.376e-03	1.253e-02	3.26e+02	3.26e+02
hsa-mir-3150	-1.669e-01	2.505e-03	1.301e-02	3.26e+02	1.52e+02
hsa-mir-2117	-1.668e-01	2.514e-03	1.301e-02	3.26e+02	6.60e+01
hsa-mir-1256	-1.665e-01	2.563e-03	1.312e-02	3.26e+02	1.03e+02
hsa-mir-940	-1.664e-01	2.583e-03	1.312e-02	3.26e+02	3.24e+02
hsa-mir-550a-1	-1.664e-01	2.586e-03	1.312e-02	3.26e+02	3.24e+02
hsa-mir-3139	-1.651e-01	2.795e-03	1.401e-02	3.26e+02	5.40e+01
hsa-mir-1307	-1.648e-01	2.844e-03	1.417e-02	3.26e+02	3.26e+02
hsa-mir-616	-1.633e-01	3.100e-03	1.526e-02	3.26e+02	3.25e+02
hsa-mir-663	-1.625e-01	3.252e-03	1.591e-02	3.26e+02	2.78e+02
hsa-mir-124-2	-1.624e-01	3.274e-03	1.592e-02	3.26e+02	4.20e+01
hsa-mir-328	-1.619e-01	3.382e-03	1.627e-02	3.26e+02	3.26e+02
hsa-mir-2278	-1.611e-01	3.546e-03	1.684e-02	3.26e+02	1.31e+02
hsa-mir-425	-1.604e-01	3.687e-03	1.731e-02	3.26e+02	3.26e+02
hsa-mir-491	-1.594e-01	3.912e-03	1.826e-02	3.26e+02	3.24e+02
hsa-mir-181a-2	-1.579e-01	4.266e-03	1.980e-02	3.26e+02	3.26e+02
hsa-mir-124-3	-1.576e-01	4.343e-03	2.005e-02	3.26e+02	4.40e+01
hsa-mir-676	-1.572e-01	4.448e-03	2.042e-02	3.26e+02	2.85e+02
hsa-mir-3663	-1.565e-01	4.609e-03	2.104e-02	3.26e+02	5.70e+01
hsa-mir-939	-1.558e-01	4.814e-03	2.185e-02	3.26e+02	3.14e+02
hsa-mir-545	-1.545e-01	5.170e-03	2.327e-02	3.26e+02	2.91e+02
hsa-mir-3132	-1.543e-01	5.231e-03	2.327e-02	3.26e+02	3.60e+01
hsa-mir-374b	-1.542e-01	5.265e-03	2.327e-02	3.26e+02	3.26e+02
hsa-mir-1249	-1.541e-01	5.290e-03	2.327e-02	3.26e+02	3.25e+02
hsa-mir-218-2	-1.541e-01	5.291e-03	2.327e-02	3.26e+02	3.26e+02
hsa-mir-3934	-1.541e-01	5.309e-03	2.327e-02	3.26e+02	3.26e+02
hsa-mir-2355	-1.540e-01	5.327e-03	2.327e-02	3.26e+02	3.26e+02

Target Gene/Attribute	Spearman Correlation	P-value	FDR (BH)	Event_SD	Event_TD
hsa-mir-1227	-1.532e-01	5.576e-03	2.423e-02	3.26e+02	1.29e+02
hsa-mir-942	-1.524e-01	5.822e-03	2.504e-02	3.26e+02	3.26e+02
hsa-mir-3128	-1.518e-01	6.023e-03	2.576e-02	3.26e+02	9.90e+01
hsa-mir-450a-1	-1.509e-01	6.356e-03	2.704e-02	3.26e+02	3.25e+02
hsa-mir-19b-2	-1.501e-01	6.635e-03	2.794e-02	3.26e+02	3.26e+02
